# WTAP-Mediated Glutaminase Splicing Bias Suppresses Ferroptosis in Hepatocellular Carcinoma

**DOI:** 10.34133/cancomm.0005

**Published:** 2026-02-19

**Authors:** Can Zhu, Ke Wu, Tong Wu, Jun Ma, Bo Ding, Nan Jiang, Keyi Du, Guomin Ju, Haiyang Xie, Chuanhui Peng, Jian Wu, Shusen Zheng

**Affiliations:** ^1^ Division of Hepatobiliary and Pancreatic Surgery, Department of Surgery, The First Affiliated Hospital, Zhejiang University School of Medicine, Hangzhou, Zhejiang, P. R. China.; ^2^ NHC Key Laboratory of Combined Multi-organ Transplantation, Key Laboratory of Organ Transplantation, Hangzhou, Zhejiang, P. R. China.; ^3^ Key Laboratory of the Diagnosis and Treatment of Organ Transplantation, Research Unit of Collaborative Diagnosis and Treatment for Hepatobiliary and Pancreatic Cancer, Chinese Academy of Medical Sciences, Hangzhou, Zhejiang, P. R. China.; ^4^ Department of Colorectal Surgery and Intestinal Transplant Center, The First Affiliated Hospital, Zhejiang University School of Medicine, Hangzhou, Zhejiang, P. R. China.

## Abstract

**Background:** Hepatocellular carcinoma (HCC), a highly aggressive malignancy with poor prognosis, is characterized by hyperactivation of the epidermal growth factor receptor (EGFR) signaling pathway. Glutaminase (GLS) is commonly overexpressed in numerous malignant tumors and acts as an oncogene to support cell growth and tumor progression, making it a target for cancer treatment. This study aimed to elucidate the underlying mechanisms of EGFR activation in driving glutaminolysis reprogramming and conferring ferroptosis resistance in HCC. **Methods:** Untargeted metabolomics, stable isotope-assisted metabolomic analysis, and RNA sequencing analysis were utilized to elucidate the mechanisms underlying glutaminolysis reprogramming upon EGFR activation. Immunoprecipitation, RNA pulldown, and dual-luciferase reporter assays were employed to examine the regulatory role of Wilms’ tumor 1-associated protein (WTAP) phosphorylation in *GLS* alternative splicing. Flow cytometry, cell viability assays, tumor-bearing mouse models, and HCC clinical specimens were used to validate the role of the AKT–WTAP–GLS axis in ferroptosis resistance and tumor progression. **Results:** Here, we demonstrated that AKT activated by EGFR signaling phosphorylated WTAP S176 and increased WTAP binding to methyltransferase-like protein 3. The enhanced interaction promoted the site-specific N6-methyladenosine (m^6^A) modification of GLS pre-mRNA, which in turn favored the alternative splicing of *GLS* toward glutaminase C (GAC) over kidney-type glutaminase. This switch led to increased glutamine utilization and glutathione/nicotinamide adenine dinucleotide phosphate (reduced form) biosynthesis, thereby alleviating ferroptosis and promoting tumor growth in mice. In addition, the levels of WTAP pS176 and GAC expression, which were mutually correlated, were positively associated with poor prognosis of patients with HCC. **Conclusions:** These findings uncover a critical mechanism by which tumor cells counteract ferroptosis by WTAP-mediated *GLS* alternative splicing under EGFR activation, highlighting the therapeutic potential of targeting the m^6^A-dependent GLS isoform switch in HCC and offering a rationale for the development of combination therapies.

## Background

Hepatocellular carcinoma (HCC), accounting for over 90% of all liver malignancies, is the third leading cause of cancer-related deaths worldwide [1–3]. Despite decades of in-depth and extensive research on novel prevention and therapeutic strategies, its prognosis remains unsatisfactory, primarily due to low response rate and high therapeutic resistance [4]. Exploring pathway dysregulations and epigenetic alterations may provide valuable insights into identifying novel targeted therapies and drug combinations with improved safety and efficacy.

Epidermal growth factor receptor (EGFR) overexpression has been observed in over 60% of HCC patients, and is positively correlated with poor differentiation, elevated proliferation activity, advanced tumor stage, intrahepatic metastasis, and reduced progression-free survival [5–8]. The activation of EGFR can mediate multiple signal transduction pathways, including phosphoinositide 3-kinase/protein kinase B (AKT), mitogen-activated protein kinase/extracellular signal-regulated kinases (ERKs), and Janus kinase/signal transducer and activator of transcription, directly influencing tumorigenesis [9]. Cellular metabolism in cancer is profoundly altered under oncogenic signaling activation to support uncontrolled tumor growth. However, the mechanisms by which metabolic alterations contribute to hyperproliferation and aggressive behavior in HCC under EGFR activation remain largely elusive.

Metabolic reprogramming, a hallmark of cancer, is characterized by enhanced glucose utilization through aerobic glycolysis (the Warburg effect) and up-regulated glutamine catabolism through glutaminolysis [10–12]. Multiple malignancies, including HCC, exhibit an oncogenetically driven addiction to glutaminolysis [13–16]. As the rate-limiting enzyme regulating glutamine metabolism, glutaminase (GLS) catalyzes the conversion of glutamine into glutamate, which in turn enters the tricarboxylic acid (TCA) cycle or serves as substrate for glutathione (GSH) synthesis [17]. Alternative usage of a 3′-exon during GLS pre-mRNA splicing results in the generation of 2 splice isoforms, glutaminase C (GAC) and kidney-type glutaminase (KGA). Accumulating evidence suggests that despite an overall increase in GLS expression in various tumor types, the GAC isoform is more highly expressed compared to KGA and associated with tumor progression [18–21]. Furthermore, the addiction to glutaminolysis has been associated with the isoform switching of GLS and a concomitant elevation in GAC expression levels [22]. These findings suggest that cancer cells may reprogram metabolism through isoform switching, enabling adaptation to tumor microenvironment changes. Thus, there is considerable interest in elucidating the intrinsic mechanisms regulating *GLS* alternative splicing in HCC and targeting this metabolic switch as a therapeutic strategy.

Post-transcriptional RNA modifications, known as epitranscriptomics, constitute a key regulatory layer in HCC [23,24]. RNA N6-methyladenosine (m^6^A) modification is closely implicated in RNA splicing, nuclear export, stability and translation, through the action of m^6^A “writers” (methyltransferase), “erasers” (demethylases), and “readers” [25]. Dysregulated m^6^A modifiers have been shown to function as oncoproteins or tumor suppressors that play essential roles in modulating tumor metabolic networks [26]. Interventions targeting m^6^A modifications have demonstrated efficacy in impairing tumor cell survival [27,28], thus offering novel approaches for cancer treatment. However, current studies investigating m^6^A-mediated cellular metabolic regulation have prioritized mRNA stability over post-transcriptional splicing regulation, leaving this critical regulatory mechanism relatively underexplored [29]. According to relevant studies, key enzymes involved in glycolysis (e.g., hexokinase 2, glucose transporter 1, and pyruvate dehydrogenase kinase) [30–32], lipid metabolism (e.g., delta 4-desaturase sphingolipid 2, fatty acid synthase, and stearoyl-CoA desaturase 1) [33–35], and GSH synthesis (e.g., solute carrier family 7 member 11 [SLC7A11]) [36] have been demonstrated to exhibit altered mRNA stability regulated by m^6^A modification. How pre-mRNA splicing and metabolism are orchestrated in an m^6^A-dependent manner in response to oncogenic stress and therapeutic interventions remains to be elucidated.

Ferroptosis is a form of regulated cell death characterized by the iron-dependent accumulation of lipid hydroperoxides [37]. Given the critical role of GSH in mediating redox homeostasis, key regulators involved in GSH metabolism, such as glutathione peroxidase 4 (GPX4), Glutamate-Cysteine Ligase Catalytic Subunit, and the cystine transporter SLC7A11, have been shown to suppress ferroptosis in cancer cells [38,39]. Currently, emerging studies are progressively uncovering the regulatory mechanisms underlying the ferroptosis-associated oxidative-reductive system in HCC [40,41]. Specifically, activating transcription factor 4 and ubiquitin-specific peptidase 8 have been revealed to up-regulate the transcriptional expression of SLC7A11 and reduce its GlcNAcylation [42,43], whereas creatine kinase B enhanced GPX4 stability through post-translational mechanisms [44]. However, HCC exhibits greater resistance to ferroptosis compared with other malignancies, such as renal cell carcinoma, thereby posing a major challenge to the clinical implementation of ferroptosis-inducing therapies [45]. Therefore, there is an unmet need to further elucidate the mechanisms underlying ferroptosis resistance in HCC.

To elucidate the underlying mechanism by which EGFR activation promotes HCC progression through metabolic reprogramming, we conducted metabolomic and transcriptomic analyses. We then employed in vitro and in vivo assays to investigate the role of m^6^A methylation in driving glutaminolysis reprogramming and conferring ferroptosis resistance upon EGFR activation. Finally, we investigated the therapeutic potential of targeting the m^6^A-dependent isoform switch for cancer treatment and explored potential combination therapies for HCC.

## Materials and Methods

### Patients and specimens

The HCC specimens and paired non-tumor tissues were obtained from The First Affiliated Hospital, Zhejiang University School of Medicine (Hangzhou, Zhejiang, China). All patients had received standard therapies after surgery. All postoperative patient specimens were histologically confirmed at our hospital to be HCC. None of the patients had undergone any prior treatments, such as local or systemic chemotherapy, radiotherapy, targeted therapy, immunotherapy, or other therapeutic interventions, before surgery. This study was conducted following the acquisition of written informed consent from all participants and was approved by the Institutional Ethics Committee in the hospital (Ethics Code: 2024-1508). Detailed clinical features of HCC patients are provided in Table S1.

### Animal studies

All procedures involving animals were conducted in accordance with the guidelines approved by the Ethics Committee for Laboratory Animals of the First Affiliated Hospital of Zhejiang University. Male BALB/c nude mice, aged 6 weeks, were obtained from the Shanghai Experimental Animal Center of the Chinese Academy of Sciences (Shanghai, China) and maintained in a specific pathogen-free environment. Mice were housed in a clean, well-ventilated environment with appropriate temperature and humidity, and humane endpoints included signs of severe distress or marked health deterioration. Euthanasia methods involved the use of injectable anesthetics, followed by cervical dislocation to ensure a humane and ethical process. HCCLM3 cells (Shanghai Institutes of Biological Sciences, Chinese Academy of Sciences, Shanghai, China), reconstituted with various Wilms’ tumor 1-associated protein (WTAP) or knock-in GLS mutants, were harvested and injected subcutaneously into the mice. Following a 6-day period after injection, mice were randomized into various treatment groups. Sulfasalazine (SAS, MCE, Shanghai, China) was prepared by dissolving in dimethyl sulfoxide (DMSO; Sigma-Aldrich, St. Louis, MO, USA) and further diluting in phosphate buffered saline (PBS), and administered intraperitoneally at a daily dose of 100 mg/kg. Imidazole ketone erastin (IKE, 30 mg/kg, MCE, Shanghai, China) was intraperitoneally injected every 3 days. Cetuximab (MCE, Shanghai, China) was intraperitoneally injected 0.5 mg twice per week. Once tumor diameters reached 5 mm, antisense oligonucleotides 5 (ASO5, Sunya Biological Technology, Hangzhou, Zhejiang, China) was delivered via intratumoral injection into the HCCLM3 xenografts. ASO (5 nmol) mixed with 3 μl of Lipofectamine 3000 (Thermo Fisher Scientific, Waltham, MA, USA) in 25 μl of Opti-MEM (Gibco, Waltham, MA, USA) was delivered every 3 days until the experimental endpoint. Tumor volumes were recorded every 6 days until the 30th day. Subsequently, the subcutaneous tumors were collected, fixed in 4% paraformaldehyde (PFA, Yeasen, Shanghai, China). The presence and characteristics of tumors were assessed through histological examination of hematoxylin and eosin (H&E, Beyotime, Shanghai, China)-stained sections.

### Immunohistochemistry

Following sectioning of paraffin-embedded tissues, dewaxing, and dehydration, antigen retrieval was conducted with citrate buffer (Beyotime, Shanghai, China) using a heat-mediated method. The sections of xenograft tumors were stained with antibodies against WTAP pS176 (1:200, generated by HUABIO, Hangzhou, Zhejiang, China), GAC (1:100, 19958-1-AR, Proteintech, Rosemont, IL, USA), 4-hydroxynonenal (4-HNE) (1:50, MA5-27570, Thermo Fisher Scientific, Waltham, MA, USA), EGFR (1:100, ab52894, Abcam, Cambridge, Britain), EGFR pY1068 (1:250, ab40815, Abcam, Cambridge, Britain), and GLS (1:100, a23189, Abclonal, Wuhan, Hubei, China), and nonspecific IgG (30000-0-AP, Proteintech, Rosemont, IL, USA) was used as a negative control. HCC tumor sections were stained with antibodies against WTAP pS176, GAC, 4-HNE, EGFR, EGFR pY1068, and GLS, with nonspecific IgG as a negative control. Sections were incubated 14 h at 4 °C with primary antibodies, then incubated with corresponding secondary antibodies for 1 h at room temperature. Tissue sections were quantitatively scored based on the percentage of positively stained cells and the staining intensity. The proportion of positive cells was designated a score of 0 (0%), 1 (0% to 1%), 2 (2% to 10%), 3 (11% to 30%), 4 (31% to 70%), or 5 (71% to 100%). Staining intensity was defined on a scale of 0 to 3, corresponding to negative, weak, moderate, and strong staining, respectively.

### Cell lines and cell culture conditions

The human HCC cell lines (HCCLM3 and Hep3B), glioma cell lines (LN229, LN18, U87, and U251), and HEK293T were procured from the Shanghai Institutes of Biological Sciences, Chinese Academy of Sciences (Shanghai, China). These cell lines were maintained in Dulbecco’s modified Eagle medium (DMEM, Gibco, Waltham, MA, USA), enriched with 10% fetal bovine serum (FBS, HyClone, Waltham, MA, USA). The cells were cultivated in a humidified environment at 37 °C with 5% CO_2_, using a Thermo Fisher Scientific incubator (Waltham, MA, USA).

### Drug treatment

A total of 3 × 10^5^ liver cancer cells was seeded in 6-cm dishes. Following 12 h of culture, DAA (50 μmol/ml, APExBIO, Houston, TX, USA) or cycloleucine (40 μmol/ml, MCE, Shanghai, China), inhibitors of m^6^A methylation, was added to each dish. After 24 h of treatment, the cells were collected for Western blotting and real-time quantitative polymerase chain reaction (RT-qPCR). To investigate whether WTAP-mediated GLS splicing influences ferroptosis, a total of 3 × 10^5^ human liver cancer cells were seeded in 6-cm dishes and treated with EGF (100 ng/ml, Abclonal, Wuhan, Hubei, China), erastin (10 μmol/l, MCE, Shanghai, China), Fer-1 (10 μmol/ml, MCE, Shanghai, China), SAS (MCE, Shanghai, China), or cystine-free medium (Gibco, Waltham, MA, USA). After 24 h, cells were harvested and subjected to Cell Counting Kit-8 (CCK-8) assay and flow cytometry.

### RNA extraction and RT-qPCR

Total RNA was extracted using the ESscience RNA-Quick Purification Kit (YiShan Biotech, Shanghai, China) and then reverse-transcribed into cDNA using the TaqMan Reverse Transcription Reagents kit (Applied Biosystems, Waltham, MA, USA). Expression of RNA was measured by SYBR Green (Takara, Kyoto, Japan) operated on the Bio-Rad QX100 Droplet Digital PCR system (Bio-Rad, Hercules, CA, USA), and relative RNA amount was calculated by the 2^ΔΔCt^ method with the normalization to GAPDH. All primers were derived from Sunya Biological Technology and summarized in Table S2.

### RNA sequencing

Total RNA extraction was performed with TRIzol reagent (Invitrogen, Carlsbad, CA, USA) as per the manufacturer’s instructions. The NanoDrop 2000 spectrophotometer (Thermo Fisher Scientific, Waltham, MA, USA) was employed to evaluate RNA purity and quantification. The Agilent 2100 Bioanalyzer (Agilent Technologies, Santa Clara, CA, USA) was used to assess RNA integrity. Sequencing libraries were then prepared using VAHTS Universal V6 RNA sequencing (RNA-seq) Library Prep Kit (Vazyme, Nanjing, Jiangsu, China) following the manufacturer’s instructions. The transcriptome sequencing and analysis were performed by OE Biotech Co., Ltd. (OE, Shanghai, China). Differential exon usage analysis was conducted with the DEXSeq (R/Bioconductor). Based on the hypergeometric distribution, Kyoto Encyclopedia of Genes and Genomes (KEGG) pathway enrichment analysis of differentially expressed genes was performed to identify significantly enriched terms using R (v 3.2.0).

### Immunoprecipitation and Western blotting analysis

Proteins were extracted from cultured cells utilizing lysis buffer (P0013F, Beyotime, Shanghai, China), subsequently subjected to immunoprecipitation (IP), and analyzed through Western blotting with specific antibodies. In summary, the cells were collected and subjected to wash 3 times with PBS. We lysed the resuspended cell pellets using 1% NP-40 lysis buffer (P0013F, Beyotime, Shanghai, China) with a 20-min incubation on ice. Then, the lysates were centrifuged and the supernatant was transferred to a new tube. About 10% of the supernatant was collected as inputs. The other supernatant was incubated with indicated antibodies around 12 h at 4 °C and mixed with protein A and protein G magnetic beads (Merck, Kenilworth, NJ, USA) for 3 h at room temperature. The IP beads underwent 3 washes with lysis buffer, after which they were subjected to Western blotting analysis. The blots were blocked using a 3% bovine serum albumin (Sigma-Aldrich, St. Louis, MO, USA), and incubated with primary antibodies and corresponding secondary antibodies. Primary antibodies used for Western blotting were GAC (1:1,000, 19958-1-AP, Proteintech, Rosemont, IL, USA), KGA (1:1,000, 20170-1-AP, Proteintech, Rosemont, IL, USA), WTAP (1:1,000, ab195380, Abcam, Cambridge, Britain), methyltransferase-like protein 3 (METTL3; 1:1,000, A8370, Abclonal, Wuhan, Hubei, China), SRSF3 (1:500, A9054, Abclonal, Wuhan, Hubei, China), YTHDC1 (1:1,000, ab259990, Abcam, Cambridge, Britain), AKT (1:1,000, #4691, CST, Danvers, MA, USA), p-AKT (1:1,000, #4060, CST, Danvers, MA, USA), tubulin (1:10,000, sc-8035, Santa Cruz Biotechnology, Dallas, TX, USA), glutathione *S*-transferase (GST) (1:1,000, sc-138, Santa Cruz Biotechnology, Dallas, TX, USA), His (1:1,000, SAB1305538, Sigma-Aldrich, St. Louis, MO, USA), thiophosphate ester (1:5,000, ab92570, Abcam, Cambridge, Britain), Flag (1:5,000, F7425, Sigma-Aldrich, St. Louis, MO, USA), HA (1:1,000, AE105, Abclonal, Wuhan, Hubei, China), and WTAP pS176 (1:200, HUABIO, Hangzhou, Zhejiang, China).

### GST pulldown

His-tagged purified protein (200 ng) was incubated with 100 ng of GST-tagged purified proteins together with GSH agarose beads (Sigma-Aldrich, St. Louis, MO, USA) in a binding buffer (50 mmol/l Tris-HCl pH 7.5, 1% Triton X-100, 150 mmol/l NaCl, 1 mmol/l dithiothreitol, 0.5 mmol/l EDTA, 100 μmol/l phenylmethylsulfonyl fluoride, 100 μmol/l leupeptin, 1 μmol/l aprotinin, 100 μmol/l Na_3_VO_4_, 100 μmol/l Na4P_2_O_7_, and 1 mmol/l NaF). The GSH agarose beads were then subjected to 3 sequential washes with binding buffer and PBS, and prepared for Western blotting.

### DNA construction and mutagenesis

PCR-amplified human *WTAP*, *GAC*, *KGA*, *EGFRvIII*, and *AKT* were cloned into pcDNA3.1-3XFlag (Cosmos Wisdom Biotechnology, Hangzhou, Zhejiang, China), pCDH-CMV-MCS-EF1-Puro-Flag (Cosmos Wisdom Biotechnology, Hangzhou, Zhejiang, China), pCDH-CMV-MCS-EF1-Puro-HA (Cosmos Wisdom Biotechnology, Hangzhou, Zhejiang, China), PGEX4T-1 (GST) (Cosmos Wisdom Biotechnology, Hangzhou, Zhejiang, China), or pColdI (His) vector. The mutations were generated using a QuikChange site-directed mutagenesis kit (Stratagene, Santa Clara, CA, USA): WTAP S176A and shRNA-resistant WTAP constructs containing A894T, C897G, and A900G. The PLKO.1 shRNA target sequence was as follows: WTAP 5′-GCAACACAACCGAAGATGACT-3′.

### RNA-immunoprecipitation and RIP-qPCR

RNA-immunoprecipitation (RIP) was carried out with the PureBinding RNA Immunoprecipitation Kit (GeneSeed, P0101, Guangzhou, Guangdong, China) with an anti-WTAP (1:40, ab195380, Abcam, Cambridge, Britain), YTHDC1 (1:50, ab259990, Abcam, Cambridge, Britain), and SRSF3 (1:40, A9054, Abclonal, Wuhan, Hubei, China) antibody. Following IP, RNA was purified and analyzed by qPCR. The primer sequences are listed in Table S2.

### RNA pulldown

RNA pulldown assays were performed utilizing the Pierce Magnetic RNA-Protein Pull-Down Kit (Thermo Fisher Scientific, Waltham, MA, USA) following the manufacturer’s standardized experimental procedures. A total of 100 pmol of biotin-conjugated *GLS* RNA probes containing either methylated or unmodified adenosine residues was incubated with 200 μg of cellular protein lysate and 50 μl of pre-equilibrated streptavidin-coated magnetic beads. After incubation for 2 h at room temperature and 5 washes, the streptavidin beads were boiled in SDS buffer and used for an immunoblot analysis.

For in vivo RNA–protein interaction studies, HCCLM3 cells were transfected with 100 pmol of biotin-labeled *GLS* RNA probes with unmethylated or mutated adenosine for 16 h. Cells were then lysed and incubated with 50 μl of streptavidin beads at room temperature for 1 h. The beads were subsequently boiled in SDS buffer and used for blotting. The sequences of RNA probes are provided in Table S3.

### Methylated RNA immunoprecipitation

The methylated RNA immunoprecipitation (MeRIP) assay was performed with the EpiQuik CUT&RUN m^6^A RNA Enrichment (MeRIP) Kit (Epigentek, Farmingdale, NY, USA) according to the manufacturer’s guidelines. Briefly, 20 μg of total RNA was isolated from HCC cells and mixed with 1 μl of m^6^A antibodies along with magnetic beads. The mix was incubated at room temperature for 2 h. The RNA fragments immobilized on the beads were subjected to enzymatic digestion and cleavage. Next, RNA binding beads capture the m^6^A RNA fragments. RNA was purified and subjected to qPCR analysis. The primer sequences are listed in Table S2.

### Dual luciferase reporter assay

We constructed the pmirGLO luciferase reporter plasmid (Addgene, Cambridge, MA, USA) containing GLS intron 14 and exon 15. Based on predictions from SRAMP and RMBase version 2.0, 5 predicted m⁶A sites within this region were mutated from adenine (A) to thymine (T) in the plasmid. The HCCLM3 and Hep3B cells were transfected with reporter plasmid with Lipofectamine 3000 (Thermo Fisher Scientific, Waltham, MA, USA) and treated with EGF (Abclonal, Wuhan, Hubei, China). Luciferase activity was detected by a Luciferase Assay System (Promega, Madison, WI, USA) and normalized according to control.

### Genomic editing

Genomic mutations in HCCLM3 and Hep3B cells were created using the CRISPR/Cas9 system as described previously [44]. sgRNAs were designed to target the genomic areas adjacent to human and human *GLS* mutation sites. sgRNA targeting sequence for GLS: 5′-TTGAACAACTAGCATTCCTT-3′. The annealed guide oligos containing overhangs were inserted into the PX458 vector (Addgene, Cambridge, MA, USA) digested by the BbsI restriction enzyme (NEB, Ipswich, MA, USA). Cells at 50% confluence were co-transfected with sgRNA and single-stranded donor oligonucleotide (ssOND) was used as a template to introduce mutations. After 24 h of transfection, the cells were trypsinized, and green fluorescent protein (GFP)-positive cells were selected by flow cytometry (CytoFLEX LX, Beckman, Indianapolis, IN, USA) and seeded in 96-well plates. Genotypic characterization was conducted through sequencing of PCR-amplified products using mutation-specific primers. The ssOND and primers used for sgRNA cloning and genomic DNA sequencing are listed in Table S4.

### In vitro kinase assay

The kinase reactions were performed as described previously [46]. In brief, GST-AKT1 was incubated with His-WTAP in 50 μl of kinase buffer (10 mmol/l HEPES [pH 7.2], 1 mmol/l EGTA, 5 mmol/l MgCl_2_, and 2 mmol/l CaCl_2_) and then incubated with 50 μmol/l ATP-γ-S (Sigma-Aldrich, St. Louis, MO, USA) at 30 °C for 30 min. The samples were alkylated with 50 mM PNBM (dissolved in 5% DMSO), incubated for 2 h at room temperature, and then subjected to sodium dodecyl sulfate polyacrylamide gel electrophoresis (SDS-PAGE) and Western blotting. Anti-thiophosphate ester antibody (1:5,000, ab92570, Abcam, Cambridge, Britain) was used to detect the phosphorylated proteins.

### Measurement of glutamate and glutamine

Glutamine and glutamate production were measured using the Glutamine Assay Kit (Abnova, Walnut, CA, USA) and the Glutamate Assay Kit (Sigma-Aldrich, St. Louis, MO, USA), respectively, according to the supplied protocols. Glutamate secretion was determined by deducting the measured glutamate concentration in the medium from the original glutamate concentration. All values were normalized according to control.

### Measurement of GLS activity

GLS activity was measured as described previously [47]. First, glutamine was switched to glutamate. HCC cell lysates (10 μg) were assayed on a 96-well plate added with 50 mmol/l Tris-acetate (pH 8.6), 2 mmol/l NAD, 20 mmol/l hydrazine (Sigma-Aldrich, St. Louis, MO, USA), 3.5 mmol/l L-glutamine (Sigma-Aldrich, St. Louis, MO, USA), and 20 mmol/l dipotassium phosphate (Sigma-Aldrich, St. Louis, MO, USA) at 37 °C for 5 min. Second, glutamate dehydrogenase was used to form αKG and NADH. We added 0.5 units of bovine L-glutamate dehydrogenase (Sigma-Aldrich, St. Louis, MO, USA) per well. NADH absorption at 340 nm was determined using Multiskan SkyHigh Full-Spectrum Microplate Reader (Thermo Fisher Scientific, Waltham, MA, USA). The assay was performed with technical triplicates.

### Cell viability detection

Assessment of cell viability was performed with a CCK-8 kit (Dojindo, Kumamoto, Japan). Cells were plated in 96-well plates at a density of 10,000 cells per well. Following a 24 h treatment with varying concentrations of SAS (MCE, Shanghai, China) or Erastin (MCE, Shanghai, China), the cells were incubated with 10 μl of CCK-8 reagent in 100 μl of DMEM per well for 1 h. The absorbance at 450 nm was determined by MD SpectraMax M5 (molecular devices, San Jose, CA, USA).

### Cell death and lipid ROS

Cell death was determined by propidium iodide (PI, Sigma-Aldrich, St. Louis, MO, USA) staining. In brief, cells were seeded into 6-cm dishes. After being treated with different reagents or cystine-free medium for specified durations on the next day, both adherent and floating cells were collected, stained with 5 μg/ml PI. Flow cytometry was used to determine the percentage of the PI-positive cell population. Lipid reactive oxygen species (ROS) was detected by BODIPY-C11 (invitrogen, Carlsbad, CA, USA). Cells were seeded in 6-cm dishes and treated the next day with the indicated reagents or cystine-free medium for specified durations. Cells were then incubated with 5 μmol/l BODIPY-C11 for 30 min at 37 °C and analyzed on CytoFLEX LX (Beckman, Indianapolis, IN, USA). Flow cytometry data were acquired using CytExpert (v.2.3) and analyzed with FlowJo (v.10).

### Measurement of GSH/GSSG

The levels of GSH and oxidized GSH (GSSG) in HCC cells were measured by a Cayman Chemical Glutathione Assay Kit (Cayman, Ann Arbor, MI, USA) following the manufacturer’s protocol. The GSSG and total GSH concentrations were calculated with a standard curve, respectively. The concentration of reduced GSH was calculated based on the following formula: reduced GSH = total GSH (GSH + 2 × GSSG) − 2 × GSSG. All experiments were carried out in triplicate.

### Measurement of NADP^+^/NADPH

Nicotinamide adenine dinucleotide phosphate (NADP^+^)/nicotinamide adenine dinucleotide phosphate (reduced form) (NADPH) levels of HCC cells were measured with an NADP^+^/NADPH assay kit (Beyotime, Shanghai, China). The supernatant was collected after centrifugation at 12,000 ×*g* for 15 min following lysis of 1 × 10^6^ cells. The supernatants were incubated with reaction buffer at room temperature for 30 min. The absorbance at 450 nm was measured using the MD SpectraMax M5 (Molecular Devices, San Jose, CA, USA). In the absence of heat treatment at 60 °C, the calculated concentration represents the total amount of NADP^+^ and NADPH in the sample (total). Following heat treatment at 60 °C, the measured concentration corresponds to the NADPH content in the sample. The ratio of NADP to NADPH in cellular samples was calculated based on the standard curve.

### Mass spectrometry analyses of proteins

In vitro AKT-phosphorylated WTAP was digested in the digestion buffer (50 mmol/l Tris-HCl [pH 8.0] and 0.5 mmol/l zinc acetate) overnight at 37 °C with 200 ng of mass spectrometry-grade AspN protease (Thermo Fisher Scientific, Waltham, MA, USA). The digested product was analyzed using liquid chromatography–tandem mass spectrometry (LC-MS/MS) on an Orbitrap Elite mass spectrometer (Thermo Fisher Scientific, Waltham, MA, USA). For identification of interacting proteins, a protein band visualized via Coomassie blue staining (Beyotime, Shanghai, China) was excised from a 10% SDS-PAGE gel and digested in gel. The digested protein samples were analyzed using high-sensitivity LC-MS/MS with an Orbitrap Elite mass spectrometer (Thermo Fisher Scientific, Waltham, MA, USA). Proteins were identified by searching the fragment spectra against the UniProt protein database (EMBL-EBI) using the Mascot search engine (v.2.3; Matrix Science) with the Proteome Discoverer software program (v.1.4; Thermo Fisher Scientific, Waltham, MA, USA).

### Metabolic measurements

For ^13^C-labeled metabolomics, 1 × 10^5^ cells were plated in 10-cm dishes in DMEM with 10% FBS. After 24 h, the culture medium was replaced with medium containing 2 mmol/l ^13^C-labeled glutamine (MCE, Shanghai, China). Cells were harvested after 6 h for subsequent LC-MS/MS. The mass isotopologues of a specific metabolite are denoted as M+0 to M+5, representing incremental mass increases resulting from ^13^C isotopic labeling.

### RG6 assay

RG6 Splicing Reporter Vector [48] was engineered to remove the first cardiac troponin T intron and to incorporate human *GLS* gene intron 14 and exon 15. Cells were seeded in 12-well plates containing one coverslip per well (Thermo Fisher Scientific, Waltham, MA, USA) 12 h prior to transfection. Transfections were performed with Lipofectamine 3000 (Thermo Fisher Scientific, Waltham, MA, USA) according to the manufacturer’s protocol. Cells transfected with the RG6 splicing reporter for 48 h were subjected to 3 PBS washes, fixation with 4% PFA, and 4′,6-diamidino-2-phenylindole (Thermo Fisher Scientific, Waltham, MA, USA) staining.

### Metabolomics

The untargeted metabolomics profiling was performed on the XploreMET platform (Metabo-Profile, Shanghai, China). As described previously [49], cells were harvested from 6-cm dishes after corresponding treatment. Metabolites were extracted in 80% cold methanol (Sinopharm Chemical Reagent Co., Ltd, Shanghai, China) followed by speed vacuum drying. Dried metabolites were reconstituted in LC-MS grade water containing 0.03% formic acid, followed by vortexing and centrifugation at 4 °C for 15 min to remove debris. Prior to LC-MS/MS analysis, samples were randomized and processed in a blinded manner. Chromatographic separation was carried out on a Nexera UHPLC system (Shimadzu, Kyoto, Japan) equipped with a reversed-phase HSS T3 column (2.1 × 150 mm, 1.8 μm; Waters, Milford, MA, USA). The gradient elution was programmed as follows: 0 to 3 min, 99% A; 3 to 15 min, 99% to 1% A; 15 to 17 min, 1% A; 17 to 17.1 min, 1% to 99% A; and 17.1 to 20 min, 99% A. Mobile phase A consisted of 0.03% formic acid in water. Mobile phase B consisted of 0.03% formic acid in acetonitrile. Chromatographic separation was performed at 0.25 ml/min, with the column maintained at 35 °C and the autosampler at 4 °C. Mass data acquisition was carried out on an AB QTRAP 6500+ triple quadrupole mass spectrometer (SCIEX, Framingham, MA, USA) operated in multiple reactions monitoring mode. The data were performed using Multi-Quant 3.0.2 (SCIEX, Framingham, MA, USA).

### Quantification of RNA m^6^A by LC-MS/MS

Purified mRNA (400 to 1,000 ng) was enzymatically digested to nucleosides with 0.5 U nuclease P1 (Sigma-Aldrich, N8630, St. Louis, MO, USA) in 20 μl of buffer containing 10 mmol/l ammonium acetate (pH 5.3) at 42 °C for 6 h. The reaction was subsequently adjusted with 2.5 μl of MES buffer (0.5 mol/l, pH 6.5) and treated with 0.5 U CIAP (Takara, 2250 A, Kyoto, Japan) for an additional 6 h at 37 °C. Mixtures were diluted to 60 μl, filtered, and analyzed by LC-MS/MS using an Agilent Poroshell 120 column coupled online to AB SCIEX Triple Quad 5500 LC mass spectrometer (Applied Biosystems, Waltham, MA, USA) in positive electrospray ionization mode [50]. The m^6^A concentrations were determined using standard curves from pure nucleoside standards, and the ratios of m^6^A/A were calculated by concentration.

### Public database analysis

The Cancer Genome Atlas (TCGA; http://www.cbioportal.org) databases were used to validate the potential roles of GLS, GAC, and KGA in liver cancer.

### Semi-quantitative RT-PCR

Total RNA was extracted from cells or tissue using TRIzol (Invitrogen, Carlsbad, CA, USA) and reverse-transcribed with Maxima H Minus Reverse Transcriptase (Thermo Fisher Scientific, Waltham, MA, USA) using oligo-dT primers. cDNA was amplified with primers for GAC and KGA, followed by separation on a 1.5% PAGE gel. ImageJ was used to measure band pixel density. Relative band intensity was assessed by calculating the ratio of the RT-PCR product from alternative splicing to that from the constitutive spliced junction.

### Statistics and reproducibility

The results are presented as the mean ± SEM as indicated and were subjected to statistical analysis using 2-tailed Student’s *t* test, 2-way analysis of variance, as appropriate. SPSS software (version 19.0, IBM, Armonk, New York, USA) was used for statistical analysis. A *P* value of <0.05 was considered statistically significant.

## Results

### EGF regulates GLS isoform expression while boosting glutaminolysis

**Fig. 1.**
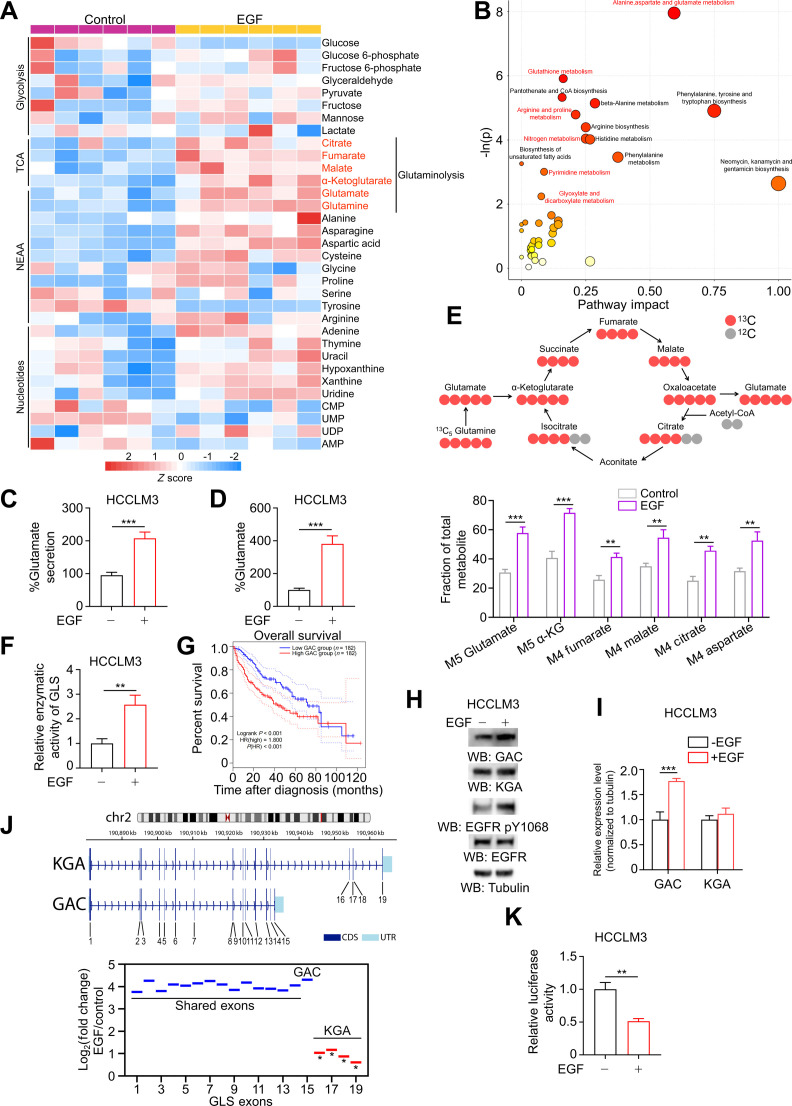
EGF regulates the expression of GLS isoforms while boosting glutaminolysis. (A) Heatmap of relative abundance of up-regulated metabolites in HCCLM3 cell treatment with 100 ng/ml EGF for 12 h (*n* = 6). (B) Pathway impact analysis showing the significantly affected metabolic pathways by EGF in HCCLM3 cells. The size of the circle represents the pathway impact, and the yellow-to-red color gradient corresponds to the −ln(P). The pathways related to glutamine and glutamate metabolism were highlighted in red. (C) HCCLM3 cells were treated with 100 ng/ml EGF for 12 h, and the culture media were collected to analyze glutamate secretion (*n* = 3). (D) HCCLM3 cells were treated with 100 ng/ml EGF for 12 h, and the cells were collected to analyze glutamate production (*n* = 3). (E) Tracing of ^13^C-labeled glutamine influx and mass spectrometry analyses of [U-^13^C_5_] glutamine-derived metabolites in HCCLM3 cells with or without 100 ng/ml EGF treatment (*n* = 3). (F) The glutaminase activity in whole cell lysates was measured in HCCLM3 cells with or without 100 ng/ml EGF treatment for 12 h. (G) Kaplan–Meier analysis of overall survival of 364 HCC patients (data obtained from TCGA). (H) EGF treatment (100 ng/ml) led to EGFR pY1068 activation and promoted GAC expression rather than KGA, as shown by Western blot. (I) The relative protein expression levels of (H), normalized to tubulin. (J) Chromosome 2 displays the *GLS* loci along with the exon structures of the *KGA* and *GAC* isoforms (top panel). Exon differential expression after 100 ng/ml EGF treatment in HCCLM3 cells (bottom panel). Asterisks denote FDR < 0.05. (K) Relative activity of the luciferase reporters in HCCLM3 cells was determined after 100 ng/ml EGF treatment for 12 h. Data are the mean ± SD, *P < 0.05; **P < 0.01; ***P < 0.001 (2-tailed Student’s *t* test). Abbreviations: EGF, epidermal growth factor; HCC, hepatocellular carcinoma; KGA, kidney-type glutaminase; GAC, glutaminase C; EGFR, epidermal growth factor receptor; TCGA, The Cancer Genome Atlas; SD, standard deviation.

EGFR signaling drives cancer cell proliferation, and its expression, amplification, and mutations have been linked to poor prognosis and therapeutic resistance in HCC [7,51]. Following our identification of the SERPINE2-EGFR axis as a potential therapeutic target in liver cancer [52], we next investigated whether EGFR activation promotes HCC progression by driving metabolic reprogramming. Specifically, a metabolite profiling analysis was performed in HCCLM3 cells treated either with or without EGF. EGF stimulation resulted in changes in the levels of many metabolites, as illustrated by heatmap alignment, including those involved in glycolysis, TCA cycle, nonessential amino acids, and nucleotides. Notably, metabolites directly associated with glutaminolysis showed particularly pronounced up-regulation following EGF treatment, compared to the broader metabolic landscape (Fig. 1A). Additionally, the pathway enrichment analysis of differentially accumulated compounds showed a significant enhancement in pathways that involve glutamine and glutamate metabolism (Fig. 1B). Next, we measured the glutamate secretion, as well as the levels of glutamine and glutamate in the cell extracts in both HCCLM3 and Hep3B cells. A marked increase in both extracellular and intracellular glutamate levels was noted after EGF treatment (Fig. 1C and D and Fig. S1A and B), accompanied by a rise in intracellular glutamine levels and a significantly elevated Glu/Gln ratio (Fig. S1C and D), suggesting that EGFR activation may boost glutaminolysis. Stable isotope-assisted metabolomic analysis using [U-^13^C_5_] glutamine as a tracer further validated that EGF significantly enhanced the levels of almost all the metabolites downstream of glutamine catabolism, including glutamate (M5), aspartate (M4), α-ketoglutarate (α-KG; M5), and other TCA cycle intermediates (Fig. 1E).

To evaluate the potential involvement of the rate-limiting enzyme of glutaminolysis in the observed increase in glutamine utilization, we measured the expression and enzymatic activity of GLS in HCC. The results indicated a positive correlation between GLS expression and EGFR phosphorylation, and patients with high GLS levels exhibited poorer survival (Fig. S1E to G). More importantly, EGF-stimulated cells exhibited significantly enhanced GLS enzymatic activity (Fig. 1F). Previous studies have reported that GLS existed as 2 isoforms generated by alternative splicing. Although they shared the same active site, GAC exhibited higher catalytic activity than KGA, making it more critical for replenishing TCA cycle intermediates [19,53]. This observation was further validated in HCC cell lines, as the relative enzymatic activity was higher in GAC-expressing cell models, demonstrating a significantly greater contribution of GAC to overall GLS activity (Fig. S1H). Additionally, patients bearing tumors with high GAC expression experienced a significantly poorer prognosis compared to those with low GAC expression (Fig. 1G). In contrast, KGA levels did not show a similar association with clinical prognosis (Fig. S1I). To further explore whether EGF stimulation affects GLS isoform levels, the protein expression of KGA and GAC were detected. While a marked elevation in GAC protein expression was observed, KGA expression remained unaltered following EGF treatment (Fig. 1H and I and Fig. S1J and K). Moreover, we transfected HCCLM3 and Hep3B cells with constitutively active variant EGFRvIII. Results showed that EGFRvIII expression increased GAC protein levels (Fig. S1L and M). Furthermore, RT-qPCR was performed to investigate the transcriptional basis of these alterations. Notably, the mRNA expression pattern reflected the protein expression levels, with the ratio of GAC/KGA markedly increasing following EGF treatment (Fig. S1N). To further assess the influence of EGFR activation on GLS pre-mRNA splicing, RNA-seq was performed. The results indicated that EGF treatment led to a dramatic increase in GAC’s exclusive exon usage, whereas the exclusive exon usage of KGA exhibited minor alterations (Fig. 1J), suggesting that EGFR activation may preferentially promote the splicing of *GLS* toward the GAC isoform. Additionally, KEGG pathway analysis also indicated that EGFR activation up-regulated pathways associated with glutamine metabolism (Fig. S1O). Based on these findings, we employed a luciferase-based reporter assay by cloning GLS intron 14 along with exon 15 (harboring the alternative splicing site) into the dual luciferase reporter vector (Fig. S1P). EGF stimulation significantly reduced luciferase activity (Fig. 1K), demonstrating that EGFR signaling promotes GLS alternative splicing by preferentially enhancing the expression of GAC isoform.

### m^6^A methylation governs the switch between GLS splicing isoforms

**Fig. 2.**
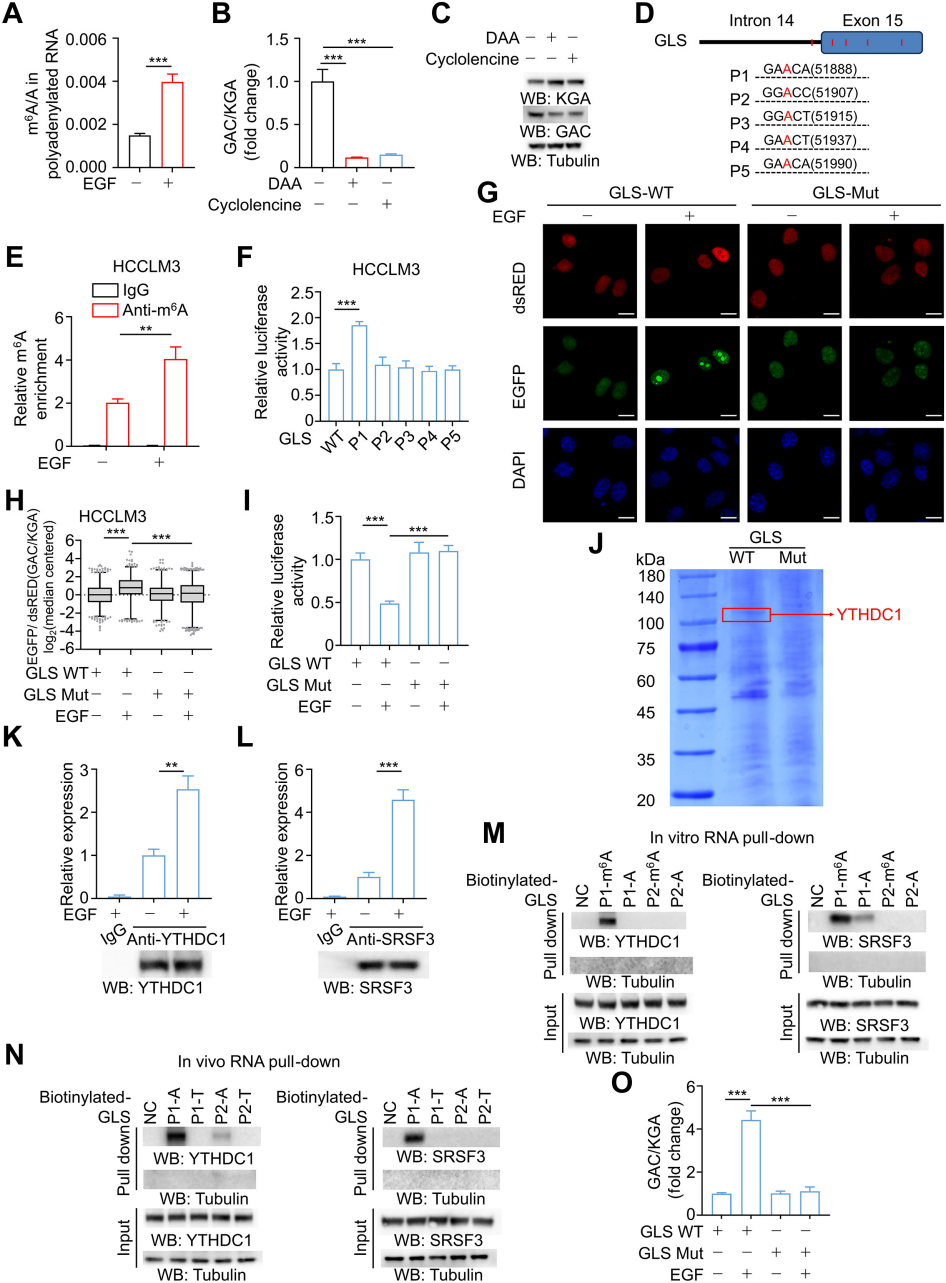
m^6^A methylation governs the switch between GLS splicing isoforms. (A) LC-MS/MS quantification of m^6^A abundance in mRNA from HCCLM3 cells with or without 100 ng/ml EGF treatment (*n* = 3). (B) The ratio of GAC/KGA in the indicated cells was detected using RT-qPCR after treatment with DAA or cycloleucine (*n* = 3). (C) DAA or cycloleucine treatment promoted KGA expression and reduced GAC expression as measured by Western blotting analysis. (D) The potential m^6^A modification sites on GLS RNA were predicted using SRAMP and RMBase v2.0. (E) MeRIP assays were conducted in HCCLM3 cells and m^6^A levels of the targeted splicing-regulatory region were measured by qPCR. (F) Relative activity of the GLS WT and Mut (P1, P2, P3, P4, and P5) luciferase reporters in EGF-treated HCCLM3 cells were determined (normalized to negative control groups; *n* = 3). (G and H) Fluorescence microscopy images (G) of HCCLM3 cells transfected with the RG6 WT and Mut reporter and the analysis of the EGFP/dsRED ratio (H). At least 1,721 independent cells evaluated for each group. (I) Relative activity of the GLS WT and Mut luciferase reporters in HCCLM3 cells was determined with or without EGF treatment (*n* = 3). (J) Coomassie Brilliant Blue stain of IP products of GLS RNA probe. (K and L) RIP-qPCR was performed to validate the interaction between GLS RNA and YTHDC1 or SRSF3 in HCCLM3 cells using the anti-YTHDC1 antibody (K) or SRSF3 antibody (L) (*n* = 3). (M and N) In vitro (M) and in vivo (N) RNA pulldown assays for YTHDF1 (left) or SRSF3 (right) binding to biotinylated GLS probes in HCCLM3 cells. Tubulin was used as the control. (O) The ratio of GAC/KGA in the indicated cells was detected using RT-qPCR. Data are the mean ± SD, **P < 0.01; ***P < 0.001 (2-tailed Student’s *t* test). Abbreviations: DAA, 3-deazaadenosine; EGF, epidermal growth factor; KGA, kidney-type glutaminase; GAC, glutaminase C; GLS, glutaminase; LC-MS/MS, liquid chromatography-tandem mass spectrometry; MeRIP, methylated RNA immunoprecipitation; WT, wild type; Mut, mutant; IP, immunoprecipitation; RIP-qPCR, RNA immunoprecipitation quantitative PCR; SD, standard deviation.

Global m^6^A levels have been observed to be higher in HCC patients compared to healthy controls, correlating with enhanced tumorigenic potential [54]. Interestingly, EGF treatment markedly up-regulated total m^6^A methylation levels in HCC cells, as measured by colorimetric assays and dot blot analysis (Fig. S2A and B). This increase was further corroborated with higher specificity by LC-MS (Fig. 2A). Given the critical role of GLS isoform switching in both cellular metabolism and oncogenesis, coupled with the well-established regulatory function of m^6^A modification in RNA splicing, we hypothesized that *GLS* alternative splicing might be modulated in an m^6^A-dependent manner.

To further assess this hypothesis, cells were treated with DAA and cycloleucine, inhibitors of methylation. The results showed that these compounds substantially down-regulated GAC isoform expression, accompanied by an up-regulation in KGA isoform expression at both the mRNA and protein levels (Fig. 2B and C and Fig. S2C). Additionally, potential m^6^A modification sites on GLS RNA were predicted using SRAMP and RMBase v 2.0, yielding 5 putative sites (P1, P2, P3, P4, and P5) adjacent to the alternative splicing junctions (Fig. 2D). The subsequent MeRIP-qPCR analysis employing primers spanning these 5 candidate sites demonstrated that EGF treatment significantly enhanced m^6^A levels across the targeted splicing-regulatory region (Fig. 2E and Fig. S2D). Through site-directed mutagenesis of the luciferase reporter plasmid, we demonstrated that mutation of the P1 site rather than the other 4 candidate sites caused a marked elevation in luciferase activity (Fig. 2F and Fig. S2E), suggesting that m^6^A modification at P1 may functionally regulate *GLS* alternative splicing. To further validate the regulatory role of this site, we introduced a bichromatic fluorescent splicing reporter RG6 incorporating the GLS intron 14 and exon 15 [48,55]. If splicing machinery acts on intron 14, it induces the splicing of an EGFP-tagged protein, indicating GAC formation; otherwise, a dsRED-tagged protein will be produced, representing KGA formation. The results demonstrated that mutation of the GLS P1 site markedly abrogated the EGF-induced elevation in the EGFP/dsRED ratio (Fig. 2G and H). Concomitantly, luciferase reporter assays confirmed this effect, collectively indicating that m^6^A methylation at P1 promotes a splicing bias toward GAC formation (Fig. 2I).

Following this lead, we performed RNA pulldown MS assay using RNA probes either harboring or lacking point mutations at the P1 site, and identified that the m^6^A reader YTHDC1 specifically bound to the GLS wild-type (non-mutated) probe (Fig. 2J). Furthermore, RIP-qPCR analysis confirmed that the binding affinity of YTHDC1 for GLS RNA was significantly enhanced following EGF stimulation, corroborating its role in *GLS* splicing regulation (Fig. 2K and Fig. S2F). Previous studies have established that YTHDC1 regulates pre-mRNA splicing by selectively recruiting the splicing factor SRSF3 to targeted RNA elements, thereby inducing differential splicing outcomes [56,57]. Notably, RIP-qPCR analysis with anti-SRSF3 antibody demonstrated a comparable increase in binding affinity upon EGF stimulation (Fig. 2L and Fig. S2G). Subsequently, in vitro and in vivo RNA pulldown assays were conducted to verify predicted interactions. The in vitro assay revealed that synthetic GLS RNA probes containing m^6^A modifications at the P1 site were able to bind YTHDC1 and SRSF3 (Fig. 2M). Additionally, a point mutation at the P1 site (A to T) dramatically abrogated the binding of both YTHDC1/SRSF3 to GLS RNA probes in vivo (Fig. 2N). Next, CRISPR-Cas9 genome-editing technology was employed to generate a GLS-Mut knock-in HCC cell lines (Fig. S2H and I). The knock-in expression of GLS-Mut abrogated the EGF-induced increase in GAC/KGA ratio (Fig. 2O). Furthermore, YTHDC1 knockdown in HCC cells similarly abolished the EGF-induced elevation of the GAC/KGA ratio (Fig. S2J and K). Lastly, the role of the m^6^A writer in regulating splicing was investigated. Among the 3 methyltransferases tested, interference with METTL3 or WTAP, but not METTL14, abrogated the effects induced by EGFR activation (Fig. S2L).

### WTAP S176 phosphorylation promotes WTAP–METTL3 binding

**Fig. 3.**
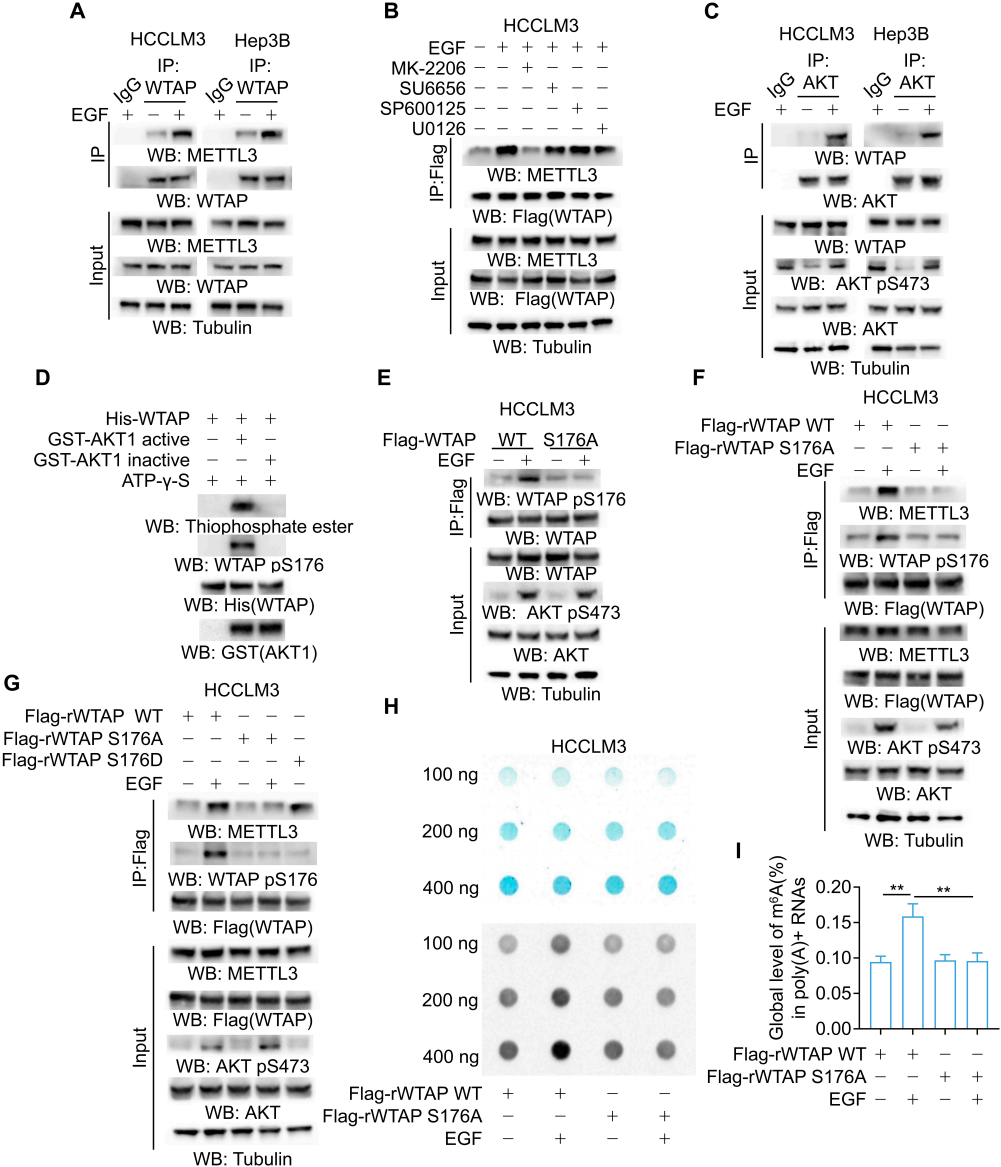
WTAP S176 phosphorylation promotes WTAP–METTL3 binding. (A) HCCLM3 and Hep3B cells were treated with or without 100 ng/ml EGF for 2 h. IP and Western blotting were performed with the indicated antibodies. (B) Flag-WTAP-transfected HCCLM3 cells were pretreated with or without MK-2206 (10 μmol/l), SU6656 (4 μmol/l), SP600125 (25 μmol/l), or U0126 (20 μmol/l) for 30 min before 100 ng/ml EGF treatment for 2 h. IP and Western blotting were performed with the indicated antibodies. (C) HCCLM3 and Hep3B cells were treated with or without 100 ng/ml EGF for 2 h. IP revealed an interaction between AKT and WTAP by Western blotting analysis. (D) An in vitro kinase assay was performed by mixing His-WTAP with GST-AKT1 active or GST-AKT1 inactive protein in the presence of ATP-γ-S. Samples were then alkylated with PNBM. (E to G) Parental HCCLM3 cells and the indicated clones with reconstituted expression of the indicated Flag-rWTAP were stimulated with or without 100 ng/ml EGF treatment. IP was performed using an anti-Flag antibody, followed by detection with Western blotting. (H) RNA m^6^A dot blot assays in HCCLM3 cells subjected to 100 ng/ml EGF treatment. Methylene blue staining served as a loading control. (I) The global m^6^A levels were quantified using an RNA methylation assay in indicated cells (*n* = 3). Data are the mean ± SD, **P < 0.01 (2-tailed Student’s *t* test). Abbreviations: WTAP, Wilms’ tumor 1-associated protein; EGF, epidermal growth factor; IP, immunoprecipitation; METTL3, methyltransferase-like protein 3; GST, glutathione *S*-transferase; His, polyhistidine; PNBM, p-nitrobenzyl mesylate; SD, standard deviation.

To further explore the mechanism by which the m^6^A methyltransferase complex coordinately regulates GLS alternative splicing under EGFR activation, HCCLM3 and Hep3B human HCC cells were stimulated with EGF, and co-IP analyses were performed. It was shown that EGF treatment enhanced the binding of WTAP to METTL3 (Fig. 3A). To delineate the mechanism by which EGF enhances the WTAP–METTL3 interaction, we pretreated HCCLM3 cells with kinase inhibitors. MK-2206, SU6656, SP600125, and U0126 were used to block the EGF-induced activation of AKT, c-Src, JNK, and ERK, respectively. Notably, only the AKT inhibitor MK-2206 abolished the EGF-induced enhancement of the WTAP–METTL3 interaction (Fig. 3B). To elucidate the precise role of AKT in the observed protein interaction, a co-IP assay was performed in HCCLM3 and Hep3B cells, and the results showed that EGF treatment enhanced the binding of WTAP to AKT (Fig. 3C). A GST pulldown assay demonstrated a direct interaction between purified bacteria-expressed GST-AKT1 and purified bacteria-expressed His-WTAP (Fig. S3A). An in vitro kinase assay revealed that purified active AKT, but not its inactive form, phosphorylated purified WTAP at the evolutionarily conserved serine (S) 176 residue in the presence of ATP-γ-S (Fig. 3D), as identified by mass spectrometry analyses (Fig. S3B and C). Furthermore, mutation of S176 into alanine (A) abrogated the purified AKT-mediated WTAP phosphorylation (Fig. S3D).

In addition, WTAP S176A, which was expressed in endogenous WTAP-depleted HCC cells (Fig. S3E), was resistant to EGF-induced WTAP phosphorylation (Fig. 3E and Fig. S3F). Together, these results demonstrated that AKT phosphorylates WTAP at S176 upon EGF treatment. To determine the effect of WTAP S176 phosphorylation on the binding of WTAP to METTL3, we conducted co-IP assays in HCCLM3 and Hep3B cells. Expression of WTAP S176A abolished the EGF-induced enhancement of WTAP binding to METTL3 (Fig. 3F and Fig. S3G), whereas the phosphomimetic WTAP S176D mutant promoted the interaction between WTAP and METTL3 in the absence of EGF treatment (Fig. 3G and Fig. S3H) [58]. To directly investigate the role of enhanced interaction between WTAP and METTL3 in the regulation of m^6^A modification, we performed m^6^A dot blot and RNA methylation quantification assay. Notably, EGF treatment markedly enhanced the m^6^A levels in HCC cells. However, this enhancement was largely abrogated upon the reconstituted expression of shRNA-resistant WTAP S176A (Fig. 3H and I and Fig. S3I). Overall, these results indicated that EGF-induced and AKT-mediated WTAP S176 phosphorylation promotes the WTAP–METTL3 interaction, thereby increasing m^6^A methylation levels in HCC cells.

### WTAP phosphorylation induces GLS splicing bias in an m^6^A-dependent manner

**Fig. 4.**
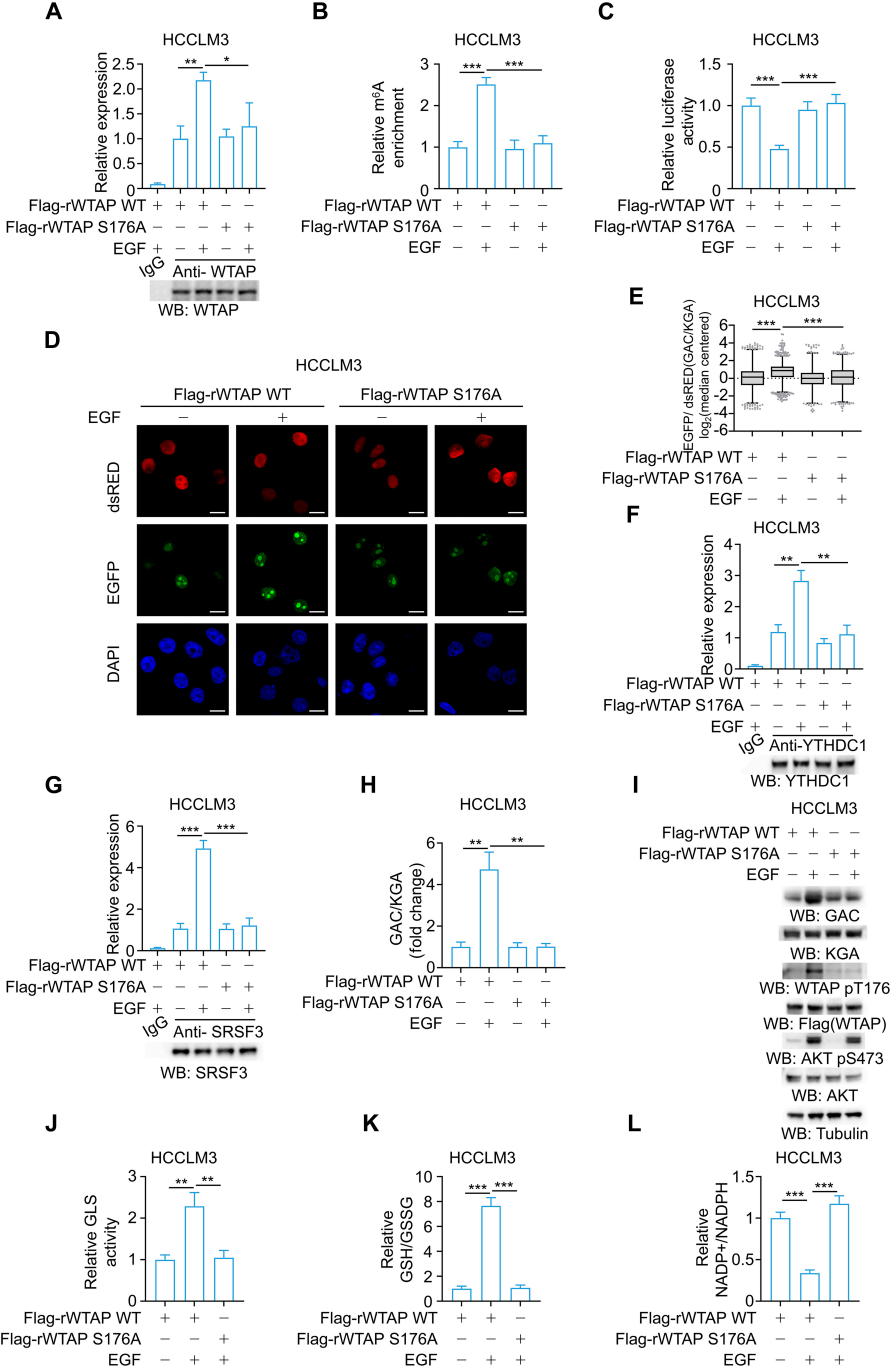
WTAP phosphorylation induces GLS splicing bias in an m^6^A-dependent manner. (A) RIP-qPCR was performed to validate the interaction between WTAP and GLS RNA in HCCLM3 cells, expressing WTAP shRNA with reconstituted expression of Flag-rWTAP WT or Flag-rWTAP S176A, using the anti-WTAP antibody (*n* = 3). (B and C) Reconstituted expression of the indicated Flag-rWTAP proteins was performed in endogenous WTAP-depleted HCCLM3 cells (*n* = 3). Enrichment of m^6^A modifications on GLS RNA was detected by MeRIP-qPCR (B). Relative activity of the luciferase reporters in HCCLM3 cells treated with 100 ng/ml EGF was determined (C). (D and E) Fluorescence microscopy images of Flag-rWTAP WT and Flag-rWTAP S176A HCCLM3 cells transfected with the RG6 splicing reporter (D) and the analysis of the EGFP/dsRED ratio (E). At least 1,578 independent cells evaluated for each group. (F and G) HCCLM3 cells expressing WTAP shRNA with reconstituted expression of the indicated WTAP proteins were treated with or without 100 ng/ml EGF (*n* = 3). RIP was performed with the anti-YTHDC1 antibody (F) or SRSF3 antibody (G). qPCR was performed to measure the relative expression of GLS. (H) After treatment with 100 ng/ml EGF, the ratio of GAC/KGA in Flag-rWTAP WT or Flag-rWTAP S176A HCCLM3 was detected using RT-qPCR. (I) After treatment with 100 ng/ml EGF, lysates of the Flag-rWTAP WT or Flag-rWTAP S176A HCCLM3 were prepared. Western blotting was performed with the indicated antibodies. (J) The GLS activity in whole cell lysates was measured in indicated HCCLM3 cells with or without 100 ng/ml EGF treatment (*n* = 3). (K and L) After treatment with 100 ng/ml EGF, ratios of GSH/GSSG (K) and NADP^+^/NADPH (L) in the Flag-rWTAP WT or Flag-rWTAP S176A HCCLM3 were determined (*n* = 3). Data are the mean ± SD, **P < 0.01; ***P < 0.001 (2-tailed Student’s *t* test). Abbreviations: WTAP, Wilms’ tumor 1-associated protein; EGF, epidermal growth factor; WT, wild type; EGFP, enhanced green fluorescent protein; dsRED, discosoma sp. red fluorescent protein; KGA, kidney-type glutaminase; GAC, glutaminase C; YTHDC1, YTH domain-containing protein 1; SRSF3, serine/arginine-rich splicing factor 3; GSH, glutathione; GSSG, glutathione disulfide; NADP^+^, nicotinamide adenine dinucleotide phosphate; NADPH, nicotinamide adenine dinucleotide phosphate (reduced form); SD, standard deviation.

Thereafter, the role of WTAP S176 phosphorylation in promoting *GLS* m^6^A methylation and regulating *GLS* alternative splicing was explored. WTAP IP followed by RT-PCR and RT-qPCR unraveled that reconstituted expression of Flag-rWTAP S176A, in contrast to expression of its WT counterpart, reversed the EGF-induced increase in the binding affinity of WTAP for GLS RNA (Fig. 4A and Fig. S4A). Furthermore, MeRIP-qPCR demonstrated that compared with cells reconstituted with expression of Flag-rWTAP WT, cells expressing Flag-rWTAP S176A failed to up-regulate GLS m^6^A methylation upon EGF treatment (Fig. 4B). Supporting the role of WTAP phosphorylation in downstream target regulation, the effects of the phosphorylation-mimicking WTAP S176D on GLS m^6^A modification were fully abolished by knock-in expression of GLS-Mut (Fig. S4B). Next, the luciferase-based splicing reporter assay showed that the inhibitory effects of luciferase activity under EGF stimulation were abrogated by reconstituted expression of Flag-rWTAP S176A, confirming the role of WTAP phosphorylation in modulating GLS alternative splicing (Fig. 4C). As further validation, the RG6 splicing reporter assay was performed, which revealed a significantly reduced EGFP/dsRED ratio in the Flag-rWTAP S176A reconstituted cells (Fig. 4D and E). The role of WTAP phosphorylation in mediating GLS m^6^A-dependent splicing was further supported by the expression of the phosphomimetic WTAP S176D mutants, whose effects in down-regulating luciferase activity were substantially abrogated by knock-in expression of GLS-Mut (Fig. S4C).

To validate the involvement of YTHDC1 and SRSF3 in mediating this effect, RIP-qPCR assays were performed. Under EGF stimulation, Flag-rWTAP WT promoted the binding of YTHDC1 and SRSF3 to GLS RNA, whereas Flag-rWTAP S176A abolished this effect (Fig. 4F and G). In contrast, the WTAP S176D phosphorylation-mimicking mutant exhibited enhanced binding affinity of both YTHDC1 and SRSF3 to GLS RNA, an effect that was similarly reversed by knock-in of GLS-Mut (Fig. S4D and E). The regulation of GLS isoform expression was observed at both mRNA and protein levels. Specifically, the results demonstrated that WTAP S176A expression largely abrogated the EGF-induced up-regulation of the GAC isoform, and the corresponding shift in the GAC/KGA ratio further corroborated these findings (Fig. 4H and I). Consistently, the WTAP S176D mutant recapitulated the EGF-induced elevation in the GAC/KGA ratio; however, this increase was abolished by GLS-Mut knock-in (Fig. S4F).

GLS-driven glutaminolysis fuels NADPH and GSH biosynthesis, thereby enhancing cellular resistance to oxidative stress [14,59]. The previous untargeted metabolomics pathway enrichment analysis identified GSH metabolism as one of the most significantly enriched pathways (Fig. 1B). In line with this finding, EGF stimulation enhanced GLS enzyme activity and elevated the GSH/GSSG ratio, while reducing NADP^+^/NADPH ratio in HCC cells, and these changes were completely abrogated by the reconstituted expression of Flag-rWTAP S176A (Fig. 4J to L). Of note, knock-in expression of GLS-Mut in HCC cells likewise abolished EGF-driven up-regulation of GLS activity and redox homeostasis (Fig. S4G and H). Collectively, these results indicated that WTAP S176 phosphorylation, by promoting GLS m^6^A methylation, plays a pivotal role in regulating GLS alternative splicing, favoring GAC over KGA, and enhancing the production of GSH and NADPH to maintain redox homeostasis.

### GLS splicing alterations drives resistance to ferroptosis in HCC cells

**Fig. 5.**
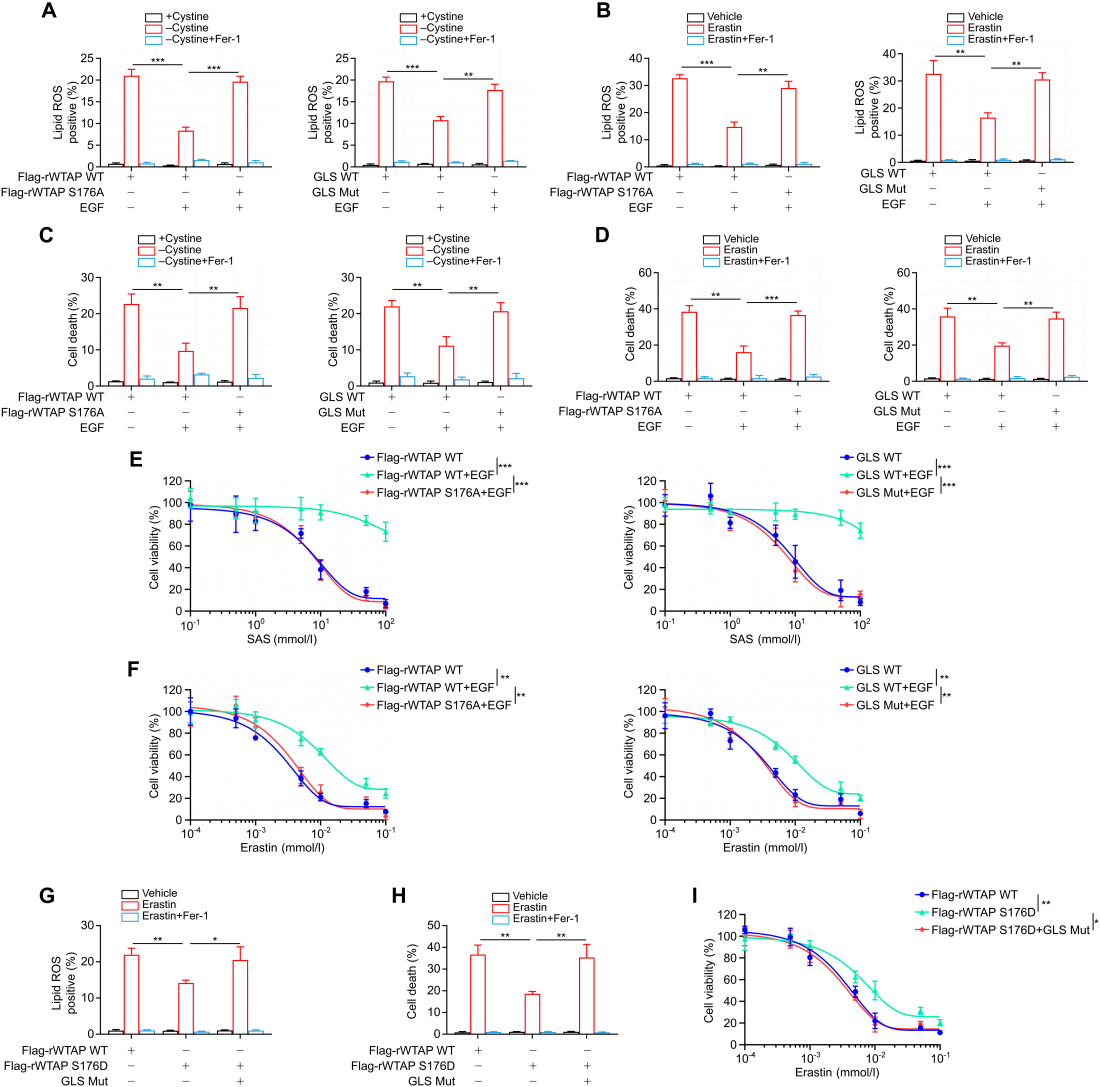
GLS splicing alterations drives resistance to ferroptosis in HCC cells. (A and B) Parental HCCLM3 cells and the indicated clones with reconstituted expression of the WTAP S176A (left) or knock-in expression of GLS Mut (right) were treated with cystine deprivation (A) or 20 μmol/l Erastin (B) and 2 μmol/l Fer-1 in the absence or presence of 100 ng/ml EGF for 24 h. Lipid ROS-positive cells were measured. (C and D) Parental HCCLM3 cells and the indicated clones with reconstituted expression of the WTAP S176A (left) or knock-in expression of GLS Mut (right) were treated with cystine deprivation (C) or 20 μmol/l Erastin (D) and 2 μmol/l Fer-1 in the absence or presence of 100 ng/ml EGF for 24 h. Cell death were measured by flow cytometry. (E and F) Parental HCCLM3 cells and the indicated clones with reconstituted expression of the WTAP S176A (left) or knock-in expression of GLS Mut (right) were treated with different concentrations of SAS (E) or Erastin (F) in the absence or presence of 100 ng/ml EGF for 24 h. Cell viability was measured by CCK-8 assay. (G to I) Parental HCCLM3 cells and the indicated clones with knock-in expression of GLS Mut were stably transfected with WTAP shRNA and reconstituted with indicated WTAP proteins. The cells were treated with or without 20 μmol/l Erastin and 2 μmol/l Fer-1 for 24 h. Lipid ROS-positive cells (G) and cell death (H) were measured by flow cytometry, respectively. The cells were treated with different doses of Erastin (I) for 24 h, cell viability was measured by CCK-8 assay. Data are the mean ± SD, *P < 0.05; **P < 0.01; ***P < 0.001 (2-tailed Student’s *t* test). Abbreviations: EGF, epidermal growth factor; KGA, kidney-type glutaminase; GAC, glutaminase C; GLS, glutaminase; WTAP, Wilms’ tumor 1-associated protein; SAS, Sulfasalazine; Fer-1, ferrostatin-1; WT, wild type; Mut, mutant; SD, standard deviation.

Given the established role of GSH in regulating redox homeostasis and its documented anti-ferroptotic function in malignancies [37,60], we speculated that the EGFR–AKT–WTAP–GLS axis confers ferroptosis resistance in HCC cells. As expected, reconstituted expression of Flag-rWTAP S176A in HCC cells abrogated EGF-induced suppression of lipid ROS production upon cystine starvation or Erastin treatment (Fig. 5A and B and Fig. S5A and B). EGF treatment ameliorated cystine starvation and Erastin-induced cell death (Fig. 5C and D and Fig. S5C and D) while increasing cell viability (Fig. 5E and F and Fig. S5E and F). More importantly, the EGF-induced ferroptosis-inhibitory effects upon cystine starvation or Erastin treatment were diminished by reconstituted expression of Flag-rWTAP S176A in HCC cells (Fig. 5A to F and Fig. S5A to F). In addition, treating cells with reconstituted expression of Flag-rWTAP S176A with Ferrostatin-1 (Fer-1), a ferroptosis inhibitor as a radical-trapping antioxidant, reduced cystine starvation and Erastin-induced lipid ROS accumulation (Fig. 5A and B and Fig. S5A and B) and cell death (Fig. 5C and D and Fig. S5C and D). Besides, EGF treatment significantly attenuated lipid ROS levels, suppressed cell death, and enhanced cell viability in cells expressing GLS-WT. Compared to their WT counterpart, knock-in expression of GLS-Mut significantly abrogated the effects induced by EGF (Fig. 5A to F and Fig. S5A to F). Of note, Fer-1 treatment in GLS-Mut-expressing cells reduced both cystine starvation and Erastin-induced lipid ROS production (Fig. 5A and B and Fig. S5A and B) and cell death (Fig. 5C and D and Fig. S5C and D).

The role of AKT-mediated WTAP S176 phosphorylation in inhibiting ferroptosis was further validated by the expression of the phosphomimetic WTAP S176D mutants, which suppressed Erastin-induced ferroptotic effects. Additionally, the protective effect elicited by WTAP S176D was attenuated by the knock-in expression of GLS-Mut (Fig. 5G to I and Fig. S5G to I). Taken together, these results indicated that activated EGFR/AKT-induced and WTAP-mediated GLS splicing alterations confer resistance to ferroptosis in HCC cells.

Given that phosphatase and tensin homolog (PTEN) loss induces constitutive AKT activation in certain glioblastoma (GBM) models [61], we investigated whether the AKT–WTAP–GLS axis confers ferroptosis resistance in PTEN-deficient GBM. Analysis of a panel of GBM cells revealed significantly higher levels of AKT S473 phosphorylation in PTEN-deficient U87 and U251 cells compared to LN229 and LN18 cells expressing wild-type PTEN. Notably, U87 and U251 cells showed increased WTAP S176 phosphorylation and GAC expression (Fig. S6A), along with enhanced resistance to Erastin-induced lipid peroxidation and cell death (Fig. S6B and C), compared to LN229 and LN18 cells.

### WTAP-mediated GLS alternative splicing promotes tumor cell growth

**Fig. 6.**
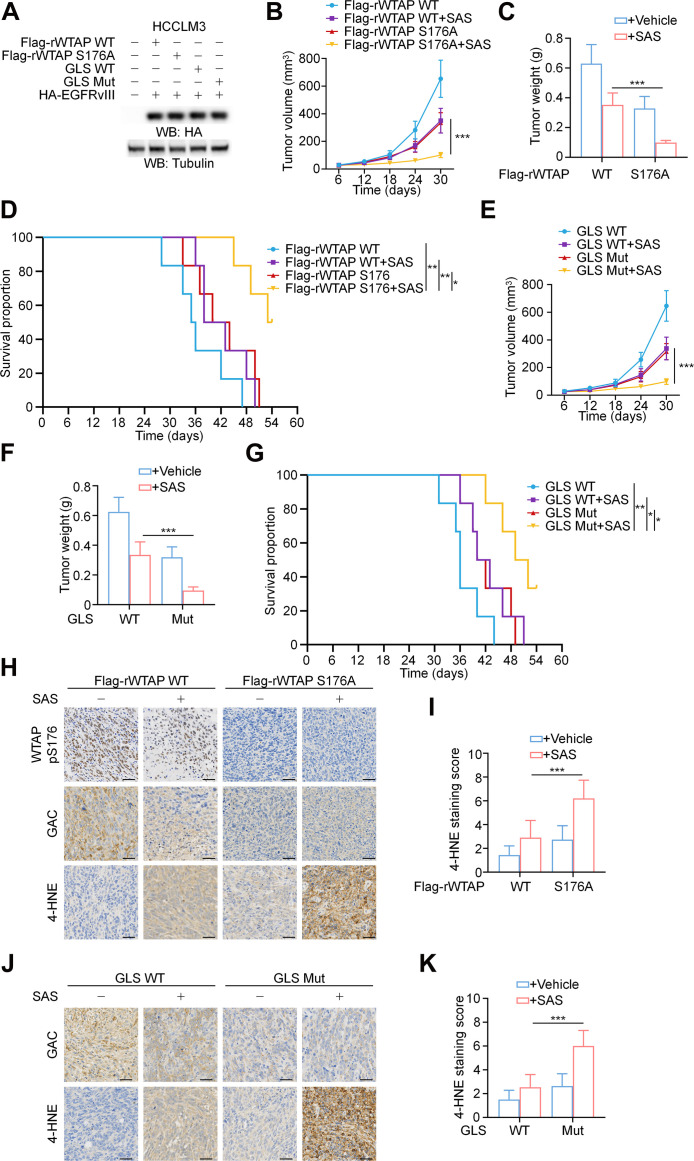
WTAP-mediated GLS alternative splicing promotes tumor cell growth. (A) The Flag-rWTAP WT, Flag-rWTAP S176A, GLS WT, and GLS Mut HCCLM3 cells expressing HA-EGFRvIII were harvested. The protein expression levels were detected by Western blotting. (B and C) EGFRvIII-overexpressing parental HCCLM3 cells and the indicated clones with expression of the WTAP S176A were subcutaneously injected into 6-week-old male athymic nude mice (*n* = 6). Upon reaching a tumor volume of 50 mm^3^, mice were randomly allocated to various treatment groups. SAS (100 mg/kg) was administered daily via intraperitoneal injection for 30 days. Tumor volumes (B) and tumor weight (C) were calculated. (D) Kaplan–Meier survival curves demonstrated significant difference between Flag-rWTAP WT, Flag-rWTAP WT +SAS, Flag-rWTAP S176A, and Flag-rWTAP S176A + SAS cohorts (*n* = 6). (E and F) EGFRvIII-overexpressing parental HCCLM3 cells and the indicated clone with knock-in expression of GLS Mut were subcutaneously injected into 6-week-old male athymic nude mice (*n* = 6). Upon reaching a tumor volume of 50 mm^3^, mice were randomly allocated to various treatment groups. SAS (100 mg/kg) was administered daily via intraperitoneal injection for 30 days. Tumor volumes (E) and tumor weight (F) were calculated. (G) Kaplan–Meier survival curves demonstrated significant difference between GLS WT, GLS WT +SAS, GLS Mut, and GLS Mut + SAS cohorts (*n* = 6). (H to K) IHC analyses of the Flag-rWTAP WT, Flag-rWTAP WT + SAS, Flag-rWTAP S176A, Flag-rWTAP S176A + SAS, GLS WT, GLS WT + SAS, GLS Mut, and GLS Mut + SAS xenograft tumors from nude mice were conducted with WTAP pS176, GAC, and 4-HNE antibodies. (H) and (J) show representative staining images from the xenograft tumors. The indicated IHC staining scores for the indicated tumor samples were compared using 2-tailed Mann–Whitney *U* test (I and K). Data are the mean ± SD, *P < 0.05; **P < 0.01; ***P < 0.001 (2-tailed Student’s *t* test). Abbreviations: EGFR, epidermal growth factor receptor; WTAP, Wilms’ tumor 1-associated protein; GLS, glutaminase; WT, wild type; Mut, mutant; SAS, sulfasalazine; IHC, immunohistochemistry; SD, standard deviation.

To investigate the role of WTAP-mediated GLS splicing bias in tumor proliferation, EGFRvIII-expressing HCCLM3 cells with or without reconstituted expression of WTAP S176A or knock-in expression of GLS-Mut were subcutaneously injected into athymic nude mice (Fig. 6A). The expression of WTAP S176A or GLS-Mut significantly inhibited tumor growth (Fig. 6B to G), accompanied by reduced GAC expression and elevated 4-HNE levels (Fig. 6H to K). Notably, treatment with SAS in combination with WTAP S176A or GLS-Mut expression produced synergistic tumor-suppressive effects, significantly improving mouse survival (Fig. 6B to G). Furthermore, this combination reduced GAC protein levels while elevating 4-HNE levels (Fig. 6H to K).

ASOs have emerged as a promising and effective approach for modulating the functions of target RNAs. The approval of several ASO-based drugs for the management of various diseases, including Duchenne muscular dystrophy and spinal muscular atrophy, further highlights the clinical potential of splice-switching ASOs [62,63]. To identify effective ASOs that promote GLS exon 15 skipping to generate KGA isoforms, the sequence surrounding the GLS P1 site was analyzed, and 6 ASOs were designed (Fig. S7A). Three ASOs (ASO1, ASO2, and ASO3) were modified with phosphorothioate linkages, while the remaining 3 (ASO4, ASO5, and ASO6) further incorporated 2′-O-methoxyethyl ribose and 5-methylcytosine modifications. Endogenous *GLS* splicing in EGFRvIII-expressing HCCLM3 cells transfected with ASOs was analyzed using RT-PCR and the findings indicated that ASO5 efficiently reduced GAC levels and increased KGA levels compared to the control (Fig. S7B). Consistently, GAC protein expression levels significantly decreased upon ASO5 treatment, whereas KGA expression levels increased (Fig. S7C). Consequently, ASO5 was selected for subsequent experiments. Notably, RT-PCR revealed a dose-dependent increase in KGA levels and a decrease in GAC levels after ASO5 treatment (Fig. S7D). Correspondingly, ASO5 treatment induced a dose- and time-dependent splicing outcome characterized by reduced GAC expression, accompanied by increased KGA expression at the protein level (Fig. S7E and F). Furthermore, ASO-mediated splicing alterations were validated using the RG6 splicing reporter assay, with ASO5 treatment demonstrating a corresponding decrease in the EGFP/dsRED ratio (Fig. S7G). As anticipated, ASO5 treatment markedly promoted Erastin-induced lipid ROS production and cell death (Fig. S7H and I). Then, a subcutaneous xenograft model was generated using EGFRvIII-expressing HCCLM3 cells, which showed that ASO5 administration, combined with SAS treatment, significantly suppressed tumor growth, down-regulated the expression of GAC, and up-regulated that of 4-HNE (Fig. S7J and K). Building on our elucidated mechanism, we further demonstrated that combining the EGFR inhibitor cetuximab with the ferroptosis inducer IKE synergistically suppressed tumor growth and significantly extended mouse survival (Fig. S8A to C). Moreover, this combination reduced GAC protein levels while elevating 4-HNE levels (Fig. S8D and E).

### WTAP pS176 and GAC predicts clinical aggressiveness of HCC

**Fig. 7.**
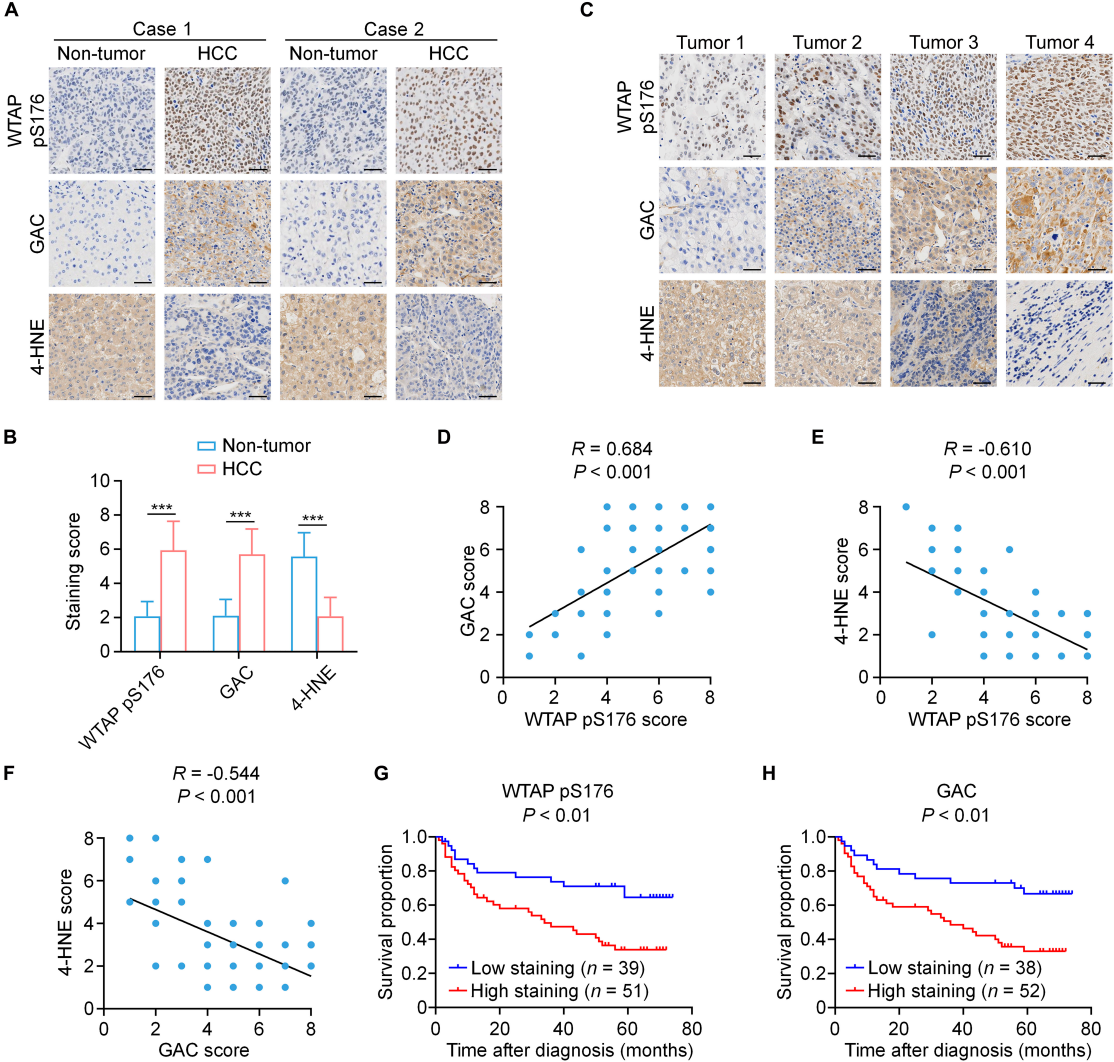
WTAP pS176 and GAC predicts clinical aggressiveness of HCC. (A and B) IHC staining was conducted on 30 human HCC specimens and their corresponding non-tumor tissues using WTAP pS176, GAC, and 4-HNE antibodies, with representative images from 2 cases presented (A). Staining scores for the target proteins in HCC and matched non-tumor liver samples (*n* = 30) were compared using 2-tailed Mann–Whitney *U* test. Data are the mean ± SD, ***P < 0.001 compared with the non-tumor adjacent tissue (B). (C to F) Human HCC samples (*n* = 60) were analyzed with WTAP pS176, GAC, and 4-HNE antibodies by IHC, with representative images presented (C). IHC staining of HCC samples using the indicated antibodies was scored, followed by correlation analyses. Statistical analysis was performed using a 2-tailed Pearson correlation test (D to F). (G and H) Kaplan–Meier survival analysis was performed to evaluate overall survival in 90 HCC patients stratified into high (staining score, 4 to 8) and low (staining score, 0 to 3) expression groups for WTAP pS176 (G) and GAC (H). *P* values were calculated using a log-rank test (2-tailed). Data are the mean ± SD, ***P < 0.001 (2-tailed log-rank test). Abbreviations: HCC, hepatocellular carcinoma; WTAP, Wilms’ tumor 1-associated protein; GAC, glutaminase C; 4-HNE, 4-hydroxynonenal; IHC, immunohistochemistry; SD, standard deviation.

To uncover the pathological significance of the AKT–WTAP–GLS axis in HCC aggressiveness, immunohistochemistry (IHC) staining was performed on 30 human HCC specimens and their adjacent normal tissues. Notably, tumor specimens exhibited elevated phosphorylation levels of WTAP S176 and higher protein expression of GAC, alongside reduced 4-HNE levels, compared with normal tissues (Fig. 7A and B). Moreover, GAC levels were positively correlated with the phosphorylation levels of WTAP S176 and inversely correlated with 4-HNE levels in HCC specimens; additionally, the phosphorylation levels of WTAP S176 were inversely correlated with 4-HNE levels (Fig. 7C to F). Next, the survival outcomes of HCC patients who had undergone standard surgical resection were evaluated. Importantly, patients with tumors exhibiting high levels of WTAP pS176 (51 cases) or GAC (52 cases) had significantly shorter survival duration than those with low levels of protein phosphorylation and expression (Fig. 7G and H). These results establish that WTAP-regulated alternative splicing of GLS drives clinical aggressiveness in human HCC.

## Discussion

**Fig. 8.**
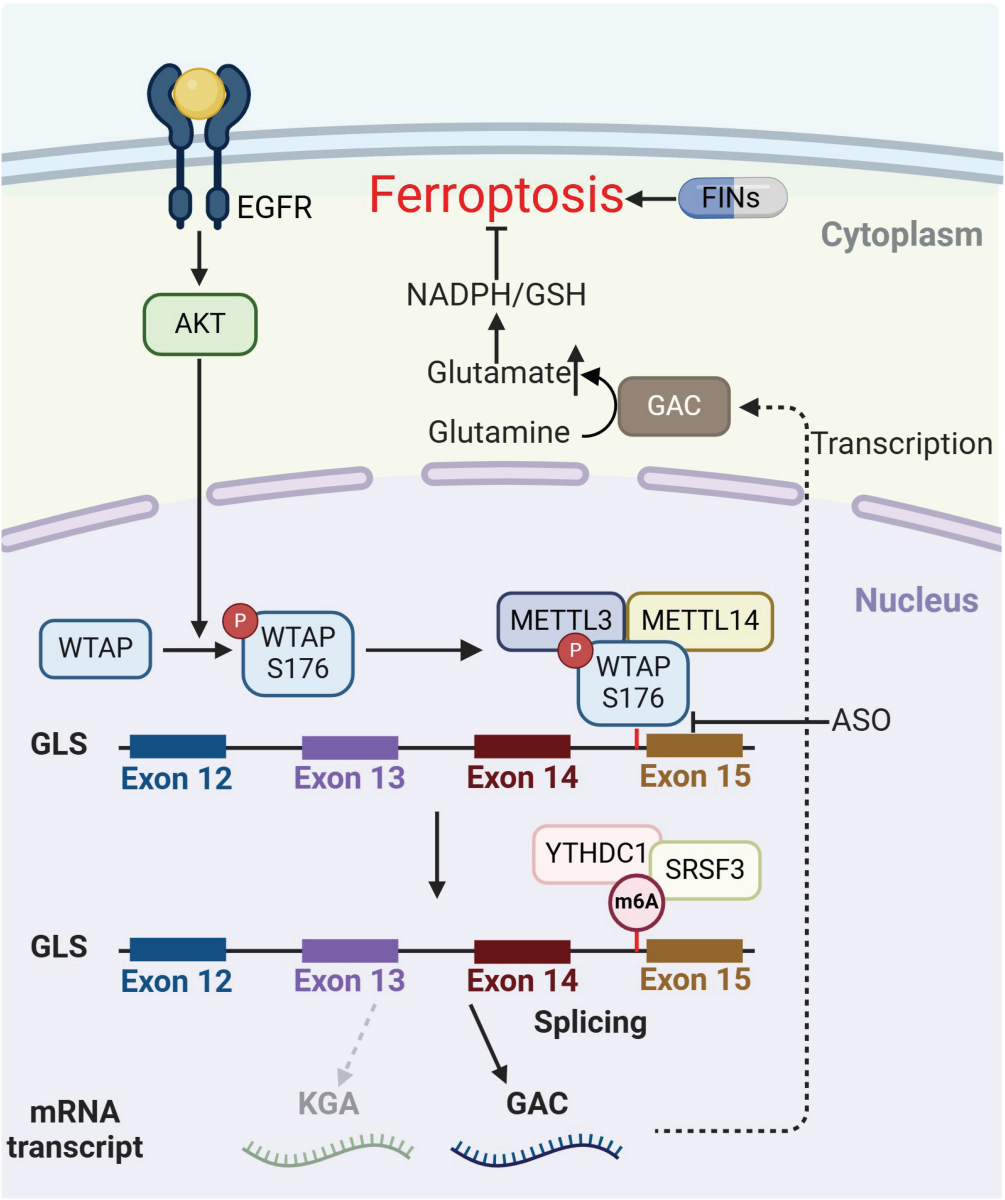
Schematic model for WTAP-mediated glutaminase splicing bias driving ferroptosis resistance in HCC. Abbreviations: EGFR, epidermal growth factor receptor; WTAP, Wilms’ tumor 1-associated protein; KGA, kidney-type glutaminase; GAC, glutaminase C; GLS, glutaminase; YTHDC1, YTH domain-containing protein 1; SRSF3, serine/arginine-rich splicing factor 3; METTL3, methyltransferase-like protein 3; METTL14, methyltransferase-like protein 14; FIN, ferroptosis-inducing agent.

Receptor tyrosine kinase activation, particularly involving EGFR signaling, is typically observed in various cancer types, including HCC [64,65]. However, its role in governing metabolic adaptation to evade ferroptosis remains unclear. Herein, we demonstrated that AKT activation promoted m^6^A-dependent alternative splicing of *GLS* through phosphorylation of WTAP, a core component of the m^6^A methyltransferase complex. Mechanistically, WTAP S176 phosphorylation enhanced its binding with METTL3, driving m^6^A methylation at the splicing-regulatory site of GLS pre-mRNA. YTHDC1 and SRSF3 were subsequently recruited to the m^6^A-modified site, which redirected the splicing machinery to favor GAC isoform generation over KGA. In turn, this enhanced the metabolic activity of GLS and concomitantly promoted the production of GSH and NADPH. It is worthwhile emphasizing that treatment with ASO reversed EGF-induced splicing alterations, while combination treatment with ferroptosis inducers exerted synergistic tumor-suppressive effects. Notably, EGF treatment alleviated the cystine starvation and Erastin-induced reductions in lipid peroxidation and cell death; this effect was abrogated by the reconstituted expression of WTAP S176A or knock-in expression of GLS-Mut. In addition, WTAP S176A or GLS-Mut greatly exacerbated the effect of SAS in inducing ferroptosis and suppressing tumor growth. These findings elucidate a regulatory axis of EGFR–AKT–WTAP–GLS in counteracting ferroptosis, highlighting the intricate interplay between epigenetic modifications, metabolic reprogramming, and ferroptosis resistance (Fig. 8).

While the functional significance of m^6^A methylation is well-characterized, gaps remain in understanding its regulation. Emerging evidence since 2015 has established that m^6^A regulatory proteins undergo dynamic post-translational regulation through acetylation, lactylation, phosphorylation, ubiquitination, and SUMOylation modifications [66]. Notwithstanding, research efforts have predominantly focused on methyltransferases, especially METTL3, with comparatively limited exploration of the remaining 2 enzymatic components. For instance, SUMO1-mediated SUMOylation of METTL3 potently inhibits its methyltransferase activity [67], while lactylation modifications in its zinc-finger domain are essential for METTL3 to capture target RNA [68]. Additionally, ERK proteins could phosphorylate METTL3 to stabilize the m^6^A methyltransferase [69], while IL-6-induced deacetylation drives METTL3 nuclear translocation to suppress cancer metastasis [70]. Herein, EGF treatment promoted the WTAP–METTL3 interaction through AKT activation. Specifically, AKT phosphorylated WTAP at the evolutionarily conserved serine (S) 176 residue in response to EGF treatment and expression of the WTAP S176A mutant in HCC cells abolished the EGF-induced increase in WTAP and METTL3 binding. Our findings establish S176 phosphorylation as a crucial regulatory modification that promotes WTAP–METTL3 interaction and offers mechanistic insights into the cooperative molecular dynamics within the m^6^A methyltransferase complex.

As the key enzyme governing glutamine consumption, GLS has been regarded as a metabolic hub driving tumor proliferation and progression through multi-layered transcriptional and posttranscriptional regulatory mechanisms. For instance, the oncogenic transcription factor c-Jun up-regulates *GLS* gene expression by directly binding to the GLS promoter [71], while c-MYC up-regulates GAC expression by transcriptionally repressing miR-23a/b, which targets the 3′ untranslated region (3′UTR) of GLS [72]. Beyond these findings, emerging evidence highlights diverse splicing regulatory mechanisms governing GLS isoform selection. Specifically, CFIm25 has been reported to play a vital role in maintaining inclusion of the GAC 3′UTR [73], whereas parallel investigations concluded that the noncoding RNA CCAT2 can modulate the function of CFIm25 at the GAC exon [55]. Furthermore, a recent study pointed out that the RNA-binding protein HuR regulates alternative splicing of GLS pre-mRNA while concomitantly modulating isoform translation and stability in breast cancer [74]. In this study, we demonstrated that WTAP S176 phosphorylation promoted GLS pre-mRNA splicing through m^6^A methylation. Additionally, the m^6^A reader YTHDC1 specifically recruited the splicing factor SRSF3 to the splicing regulatory site, mechanistically linking epitranscriptomic modification to GAC-biased alternative splicing. Our study identified a dynamic switch between KGA and GAC, triggered by WTAP phosphorylation, thus providing critical insights into the mechanism underlying post-transcriptional regulation of GLS and exemplifying the layered regulatory network that metabolic enzymes have evolved to meet fluctuating cellular environments and oncogenic signals.

Ferroptosis represents a distinct form of regulated cell death, with many of its physiological functions remaining undefined [75]. Elucidating its genotype-selective mechanisms and associated mechanisms in cancer is crucial for guiding ferroptosis-based therapeutic interventions [76]. Our study demonstrated that activated EGFR/AKT signaling induces WTAP S176 phosphorylation, which inhibited ferroptosis in HCC cells by up-regulating GAC expression to promote GSH and NADPH production. This finding expands the current understanding of ferroptosis in cancer and establishes a link between EGFR signaling, RNA m^6^A modification, GLS isoform regulation, and ferroptosis resistance, which might generate opportunities for diagnostic advancements and potential combination therapies.

Analyses of human HCC specimens compared to adjacent normal tissues revealed that tumor cells have reduced ferroptosis, with correspondingly up-regulated WTAP S176 phosphorylation and GAC expression. These findings indicated that oncogenic signaling elicits tumor-specific regulation to alleviate lipid peroxidation and counteract ferroptosis by WTAP activation-associated splicing bias of *GLS*. Such splicing alterations provide an opportunity to develop transformative therapies through the disruption of aberrant pre-mRNA splicing. To this end, splice-switching ASOs were developed to specifically target the splicing regulatory site, thereby reducing the generation of the more enzymatically active GAC isoform. Moreover, we explored the therapeutic potential of combining ferroptosis inducers with either ASOs or cetuximab in vivo. The results demonstrated marked synergistic antitumor efficacy, indicating that these combinations specifically enhanced the sensitivity of tumor cells to ferroptosis inducers and represent a promising strategy for broadening the applicability of ferroptosis inducers in HCC. Overall, this study emphasizes the critical role of oncogenic signaling-reprogrammed metabolic enzymes in conferring ferroptosis resistance.

The present study has some limitations. To begin, the employed subcutaneous xenograft model does not fully recapitulate the tumor microenvironment of human HCC. Validation in orthotopic models would enhance physiological relevance. Additionally, our mechanistic insights into the EGFR–AKT–WTAP–GLS axis could be further substantiated using conditional knock-in/knock-out mouse models. Finally, the study primarily relied on preclinical models. Although we demonstrated promising efficacy of cetuximab–IKE combination therapy in animal experiments, these findings necessitate validation in larger clinical cohorts and prospective clinical trials. Similarly, the clinical applicability and safety profile of our designed ASO-based therapy for HCC need to be confirmed by further clinical studies.

## Conclusion

This study demonstrates that upon EGFR activation, WTAP phosphorylated by AKT at S176 enhances its binding to METTL3 and promotes the m^6^A modification-dependent *GLS* alternative splicing, thereby inducing the generation of GAC. This altered GLS splicing event subsequently promotes the production of GSH and NADPH, thereby suppressing ferroptosis and enhancing tumor proliferation. In addition, the levels of WTAP pS176 and GAC are associated with poor prognosis in HCC patients. Taken together, this study elucidates the mechanistic involvement of m^6^A modification-regulated metabolic enzyme in modulating ferroptosis resistance in HCC.

## Ethical Approval

All tissue samples were obtained from patients with liver cancer who underwent surgery at the First Affiliated Hospital of Zhejiang University School of Medicine. Written informed consent was obtained from each patient. This study was approved by the Ethics Committee of the First Affiliated Hospital of Zhejiang University School of Medicine (Ethics Code 2024-1508). All animal experiments were approved by the Animal Care Committee of Zhejiang University (Ethics Code 2024-1712) and were conducted in strict accordance with the National Institutes of Health Animal Care and Use Guidelines.

## Data Availability

The data generated in this study are available within the article and the Supplementary Materials. The data that support the findings of this study are available from the corresponding authors on reasonable request. The sequencing data have been deposited in NCBI (PRJNA1354788).
