# Supplementary material for: WTAP-Mediated Glutaminase Splicing Bias Suppresses Ferroptosis in Hepatocellular Carcinoma
Source: Cancer Commun (Lond). 2026 Feb 19;46:0005. doi: 10.34133/cancomm.0005 (PMC12917114; doi:10.34133/cancomm.0005)
Supplement: Supplementary 1 — Figs. S1 to S8 Tables S1 to S4 [file cancomm.0005.f1.docx]

**Supplementary Materials**

**WTAP-mediated glutaminase splicing bias suppresses ferroptosis in hepatocellular carcinoma**

Can Zhu^1,2,3,†^, Ke Wu^2,3,4,†^, Tong Wu^1,2,3,†^, Jun Ma^1,2,3^, Bo Ding^1,2,3^, Nan Jiang^1,2,3^, Keyi Du^1,2,3^, Guomin Ju^1,2,3^, Haiyang Xie^1,2,3^, Chuanhui Peng^1,2,3^, Jian Wu^1,2,3,*^, Shusen Zheng^1,2,3,*^

^1^Division of Hepatobiliary and Pancreatic Surgery, Department of Surgery, The First Affiliated Hospital, Zhejiang University School of Medicine, Hangzhou, Zhejiang, P. R. China.

^2^NHC Key Laboratory of Combined Multi-organ Transplantation, Key Laboratory of Organ Transplantation, Hangzhou, Zhejiang, P. R. China.

^3^Key Laboratory of the Diagnosis and Treatment of Organ Transplantation, Research Unit of Collaborative Diagnosis and Treatment for Hepatobiliary and Pancreatic Cancer, Chinese Academy of Medical Sciences, Hangzhou, Zhejiang, P. R. China.

^4^Department of Colorectal Surgery and Intestinal Transplant Center, the First Affiliated Hospital, Zhejiang University School of Medicine, Hangzhou, Zhejiang, P. R. China.

^†^Can Zhu, Ke Wu, and Tong Wu contributed equally to this study.

^*^Corresponding authors:

Shusen Zheng (shusenzheng@zju.edu.cn) and Jian Wu (drwujian@zju.edu.cn); Division of Hepatobiliary and Pancreatic Surgery, Department of Surgery, The First Affiliated Hospital, Zhejiang University School of Medicine, Hangzhou 310003, Zhejiang, P. R. China.


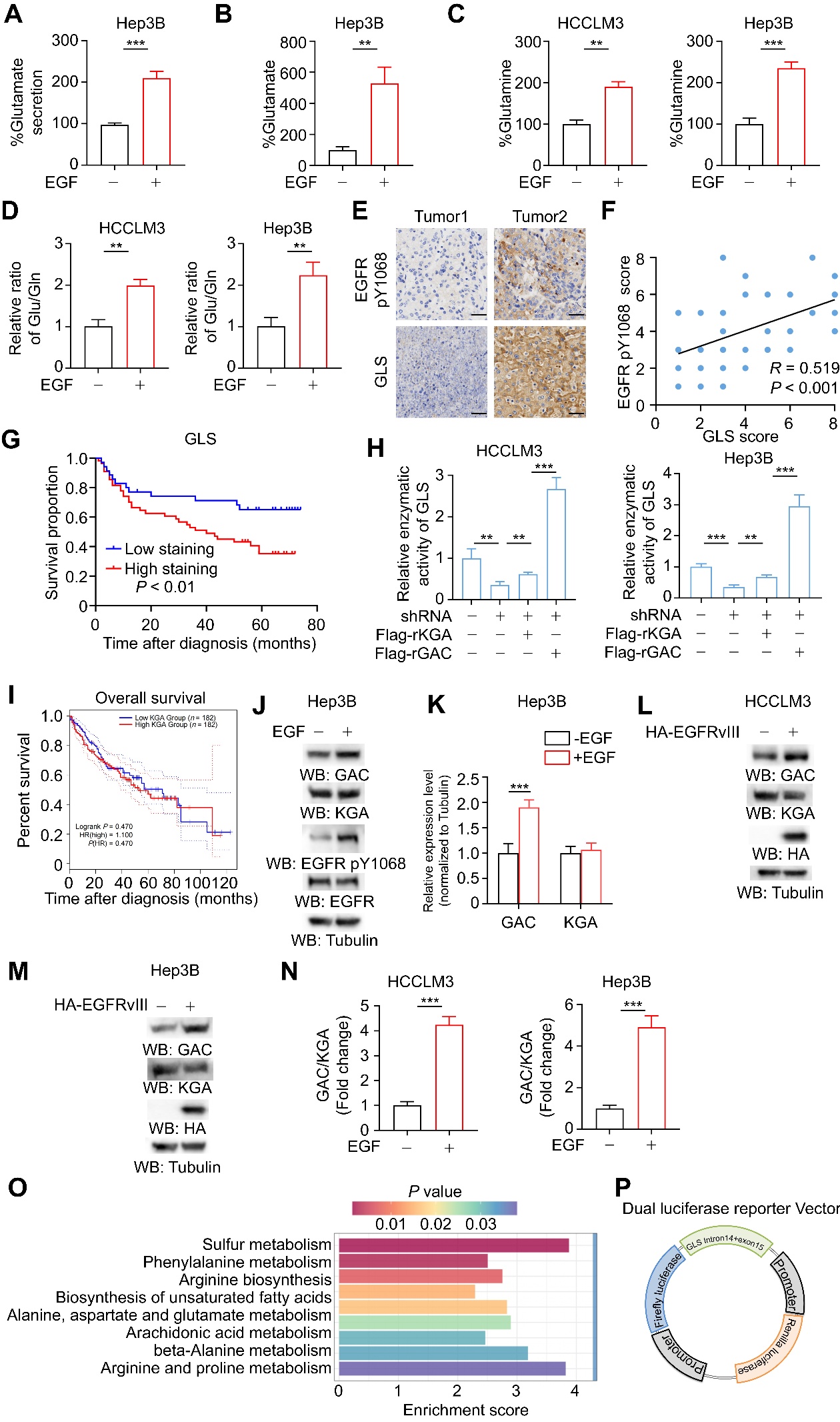


**Supplementary Figure S1.** **EGF regulates the expression of GLS isoforms while boosting glutaminolysis.**

(A) Hep3B cells were treated with 100 ng/mL EGF for 12 h, and the culture media were collected to analyze glutamate secretion (*n* = 3).

(B-D) HCC cells were treated with 100 ng/mL EGF for 12h, and the cells were collected to analyze glutamate production (B), glutamine production (C) and relative ratio of Glu/Gln (D) (*n* = 3).

(E-F) Human HCC samples (*n* = 50) were analyzed with EGFR pY1068 and GLS antibodies, with representative images presented (E). Scoring of IHC staining in HCC samples using the indicated antibodies was followed by correlation analyses (F). A Pearson correlation test was used (two-tailed).

(G) Kaplan-Meier survival analysis was performed to evaluate overall survival in 90 HCC patients stratified into high (staining score, 4-8) and low (staining score, 0-3) expression groups for GLS. *P* values were calculated using a log-rank test (two-tailed).

(H) The GLS activity of KGA and GAC were measured in HCC cells transfected with GLS shRNA, Flag-rGAC, Flag-rKGA (*n* = 3).

(I) Kaplan-Meier survival analysis was performed to evaluate overall survival of 364 HCC patients stratified into high and low KGA group (data obtained from TCGA).

(J) EGF treatment (100 ng/mL) led to EGFR pY1068 activation and promoted GAC expression rather than KGA, as shown by western blotting analysis.

(K) The relative protein expression levels of (J), normalized to Tubulin (*n* = 3).

(L-M) Expression of HA-EGFRvIII promoted GAC expression without affecting KGA expression in HCCLM3 (L) and Hep3B (M) as measured by western blotting analysis using antibodies targeting GAC, KGA, HA, and Tubulin.

(N) The ratio of GAC/KGA in the indicated cells was detected using RT-qPCR (*n* = 3).

(O) KEGG pathway analysis using RNA-seq data.

(P) Schematic illustration of the construction of dual luciferase reporter assays.

Abbreviations: HCC, hepatocellular carcinoma; EGFR, epidermal growth factor receptor; KGA, kidney-type glutaminase; GAC, glutaminase C; GLS, glutaminase; IHC, immunohistochemistry; Glu, glutamate; Gln, glutamine; RT-qPCR, reverse transcription quantitative PCR; KEGG, Kyoto Encyclopedia of Genes and Genomes.


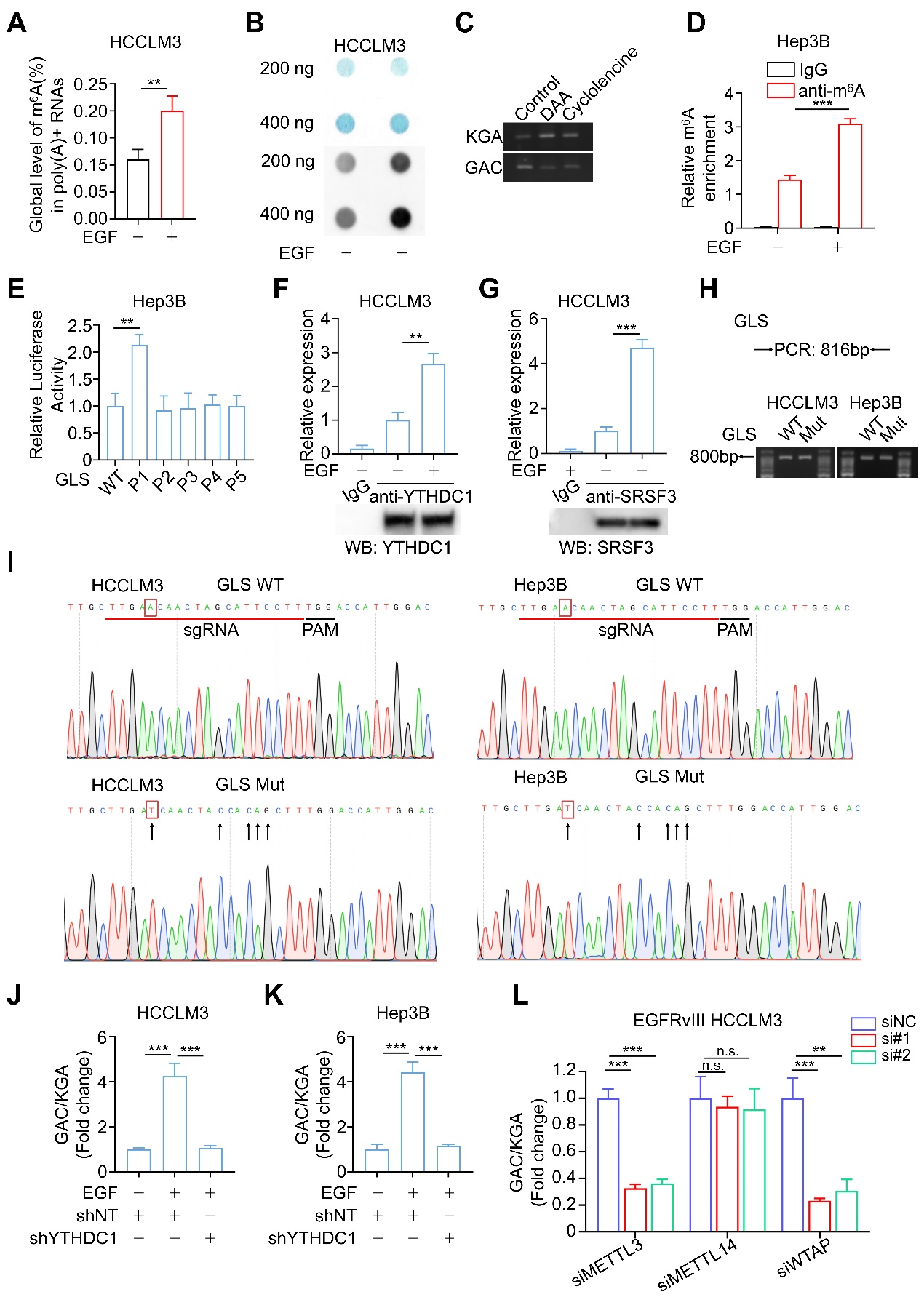


**Supplementary Figure S2.** **m^6^A methylation govern****s the switch between GLS splicing isoforms.**

(A) The global m^6^A levels were quantified using an RNA methylation quantification assay in HCCLM3 treated with 100 ng/mL EGF, based on a standard curve (*n* = 3).

(B) The m^6^A level of poly(A) + RNAs isolated from total RNA of HCCLM3 cells treated with 100 ng/mL EGF was indicated by m^6^A dot blot. Methylene blue staining served as a loading control.

(C) RT-PCR analysis of GAC and KGA in response to DAA or cycloleucine.

(D) MeRIP assays were conducted in Hep3B cells treated with 100 ng/mL EGF and m^6^A levels of the targeted splicing-regulatory region were measured by qPCR (*n* = 3).

(E) Relative activity of the GLS WT and Mut (P1, P2, P3, P4 and P5) luciferase reporters in 100 ng/mL EGF-treated Hep3B cells was determined (normalized to negative control groups, *n* = 3).

(F-G) RIP-qPCR was performed to validate the interaction between GLS RNA and YTHDC1 or SRSF3 in HCCLM3 cells using the anti-YTHDC1 antibody (F) or SRSF3 antibody (G) (*n* = 3). Western blotting analysis of YTHDC1 (F) and SRSF3 (G), which were used as internal controls for the RIP assays.

(H-I) Genomic DNA was extracted from the indicated cells. Amplified PCR products were shown (H) and sequenced (I).

(J-K) The ratio of GAC/KGA was detected by RT-qPCR in the HCCLM3 (J) and Hep3B (K) after transfection with shNT or shYTHDC1 and treatment with 100 ng/mL EGF (*n* = 3).

(L) Assessment of GAC/KGA ratio via RT-qPCR following the inhibition of METTL3, METTL14, or WTAP in EGFRvIII HCCLM3 cells (*n* = 3).

Data are the mean ± SD. ***P* < 0.01; ****P* < 0.001; n.s., not significant (two-tailed Student’s *t*-test).

Abbreviations: EGF, epidermal growth factor; DAA, 3-deazaadenosine; GLS, glutaminase; KGA, kidney-type glutaminase; GAC, glutaminase C; WT, wild type; Mut, mutant; YTHDC1, YTH domain-containing protein 1; SRSF3, serine/arginine-rich splicing factor 3; RIP-qPCR, RNA immunoprecipitation quantitative PCR; MeRIP, methylated RNA immunoprecipitation; RT-PCR, reverse transcription PCR; SD, standard deviation.


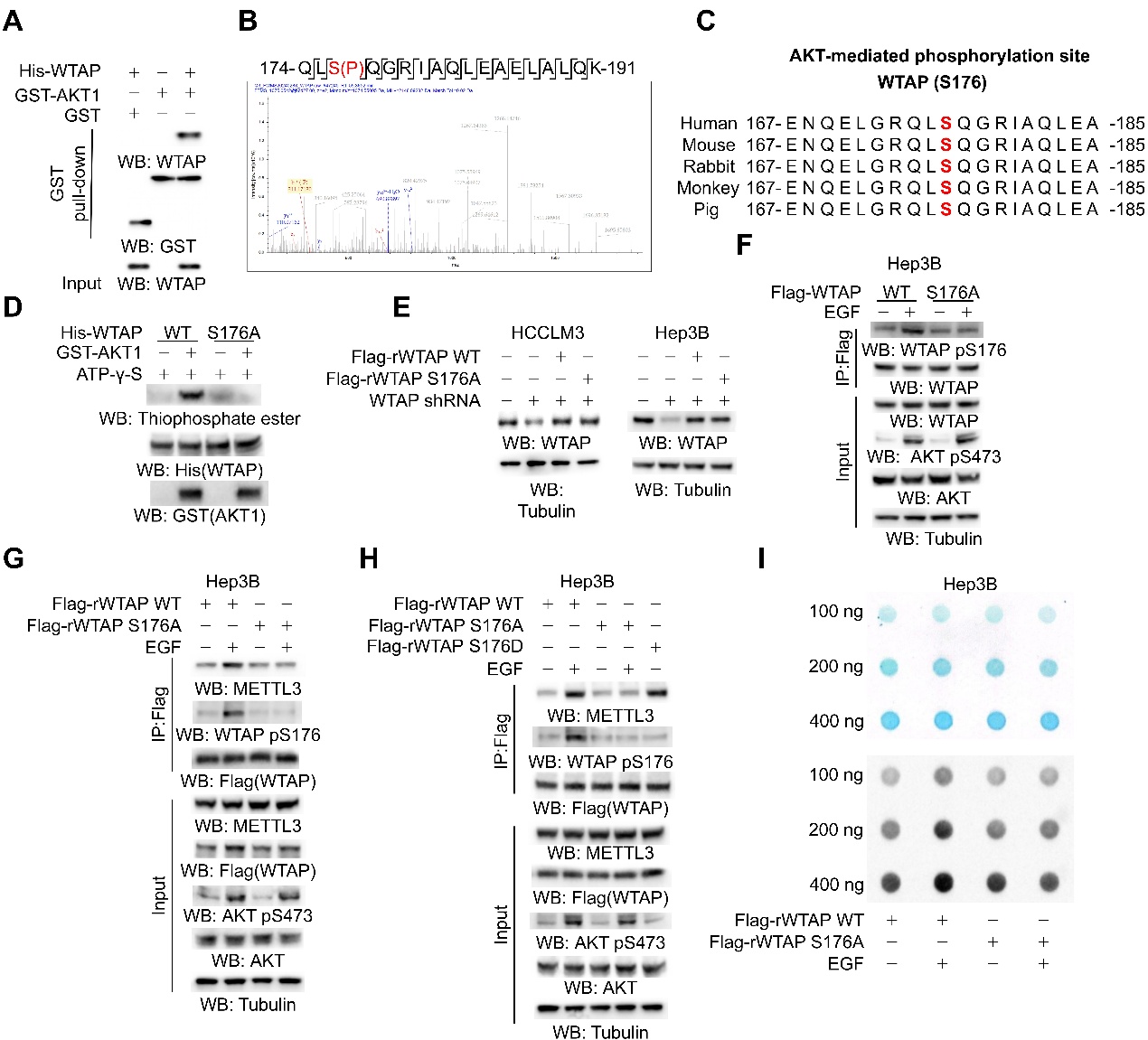


**Supplementary Figure S3.** **WTAP S176 phosphorylation promotes WTAP-METTL3 binding.**

(A) A GST pulldown assay was performed in an in vitro system using purified GST, GST-AKT1 and His-WTAP.

(B) Purified His-WTAP proteins were incubated with GST-AKT1 for an in vitro kinase assay. Mass-spectrometric analysis was performed.

(C) Alignment of protein sequences spanning WTAP S176 from different species.

(D) An in vitro kinase assay was performed by mixing GST-AKT1 with His-WTAP WT or His-WTAP S176A inactive protein in the presence of ATP-γ-S.

(E) The indicated cells expressing WTAP shRNA with reconstituted expression of the indicated WTAP proteins were harvested and analyzed by western blotting to verify WTAP expression levels.

(F-H) Parental Hep3B cells and the indicated clones with reconstituted expression of the indicated Flag-rWTAP were stimulated with or without 100 ng/mL EGF. IP was performed using the anti-Flag antibody, followed with analysis via western blotting.

(I) RNA m^6^A dot blot assays in Hep3B cells subjected to 100 ng/mL EGF treatment. Methylene blue staining served as a loading control.

Abbreviations: WTAP, Wilms’ tumor 1-associated protein; EGF, epidermal growth factor; IP, immunoprecipitation; METTL3, methyltransferase-like protein 3; GST, Glutathione S-Transferase; His, Polyhistidine.


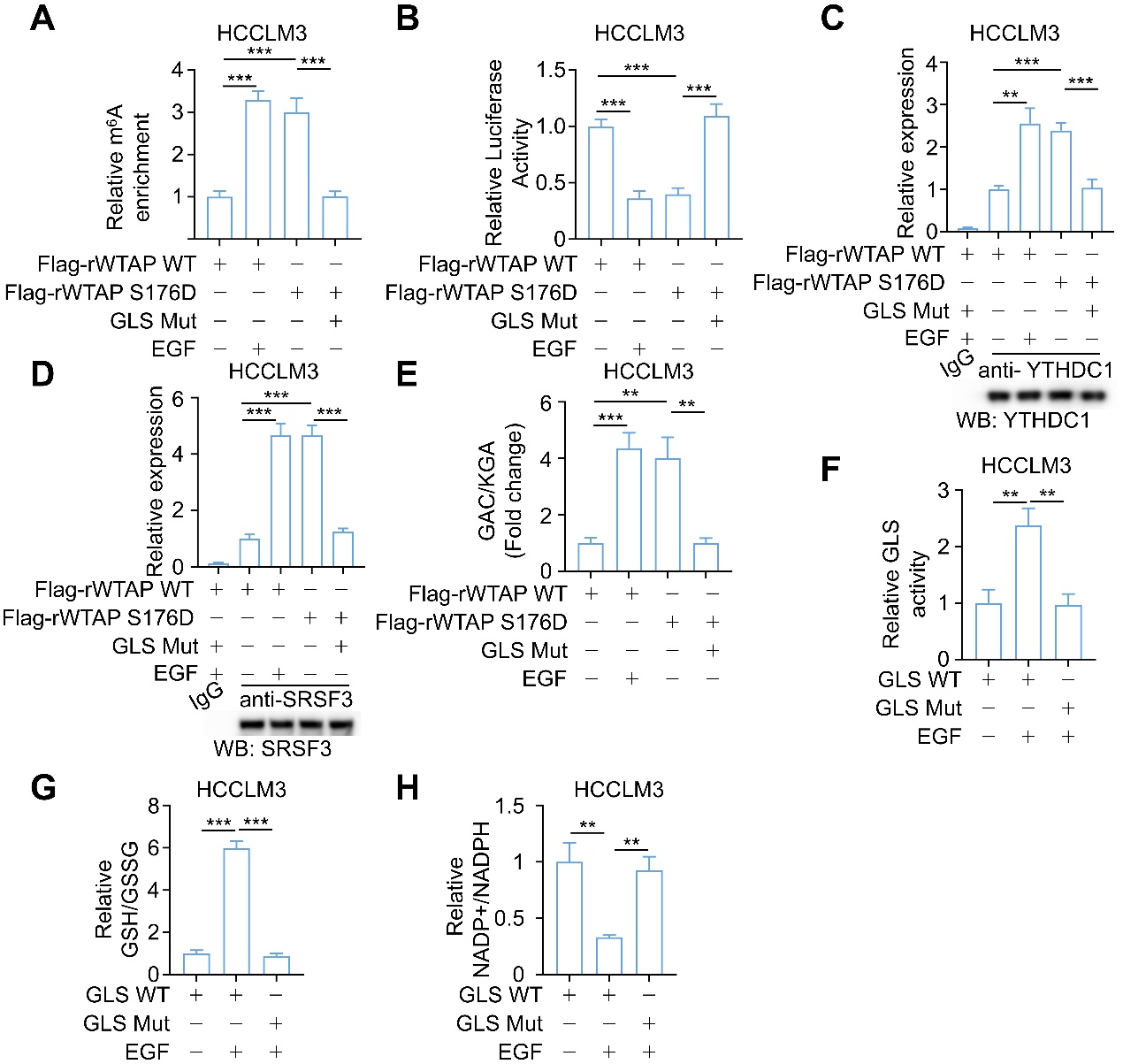


**Supplementary Figure S4. WTAP phosphorylation induces GLS splicing bias in an m^6^A -dependent manner.**

(A-B) Reconstituted expression of the indicated Flag-rWTAP proteins was performed in endogenous WTAP-knockdown HCCLM3 cells with knock-in expression of GLS Mut. Enrichment of m^6^A modifications on GLS RNA were detected by MeRIP‐qPCR (A). Relative activity of the luciferase reporters in HCCLM3 cells treated with 100 ng/mL EGF was determined (B).

(C-D) Flag-rWTAP WT and S176A HCCLM3 cells, as well as Flag-rWTAP S176A HCCLM3 with knock-in expression of GLS Mut were treated with or without 100 ng/mL EGF (*n* = 3). RIP was performed with the anti-YTHDC1 antibody (C) or SRSF3 antibody (D). qPCR was performed to measure the relative expression of GLS.

(E) After treatment with 100 ng/mL EGF, the ratio of GAC/KGA was detected using RT-qPCR in Flag-rWTAP WT and S176A HCCLM3 cells, as well as in Flag-rWTAP S176A HCCLM3 with knock-in expression of GLS Mut (*n* = 3).

(F) The GLS activity in whole cell lysates was measured in indicated HCCLM3 cells with or without 100 ng/mL EGF treatment (*n* = 3).

(G-H) Ratios of GSH/GSSG (G) and NADP^+^/NADPH (H) in GLS WT and GLS Mut HCCLM3 treated with 100 ng/mL EGF were determined (*n* = 3).

Data are the mean ± SD, ***P* < 0.01; ****P* < 0.001 (two-tailed Student’s *t*-test).

Abbreviations: WTAP, Wilms’ tumor 1-associated protein; EGF, epidermal growth factor; GLS, glutaminase; KGA, kidney-type glutaminase; GAC, glutaminase C; YTHDC1, YTH domain-containing protein 1; SRSF3, serine/arginine-rich splicing factor 3; WT, wild type; Mut, mutant; GSH, glutathione; GSSG, glutathione disulfide; NADP^+^, nicotinamide adenine dinucleotide phosphate; NADPH, nicotinamide adenine dinucleotide phosphate (reduced form); SD, standard deviation.


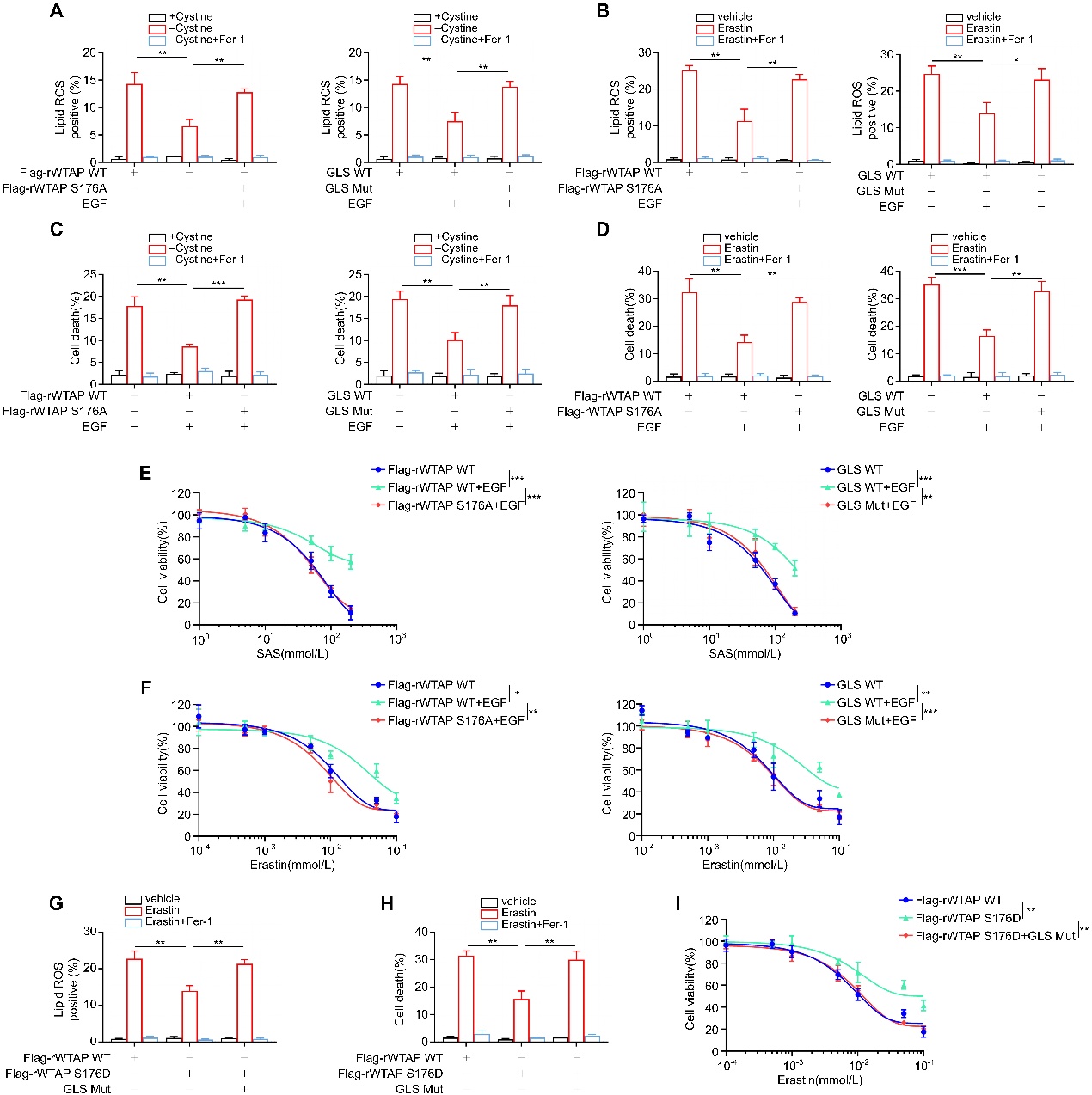


**Supplementary Figure S5. GLS splicing alterations drives resistance to ferroptosis in HCC cells.**

(A-B) Parental Hep3B cells and the indicated clones with reconstituted expression of the WTAP S176A (left) or knock-in expression of GLS Mut (right) were treated with cystine deprivation (A) or 20 μmol/L Erastin (B) and 2 μmol/L Fer-1 in the absence or presence of 100 ng/mL EGF for 24 h. Lipid ROS-positive cells were measured.

(C-D) Parental Hep3B cells and the indicated clones with reconstituted expression of the WTAP S176A (left) or knock-in expression of GLS Mut (right) were treated with cystine deprivation (C) or 20 μmol/L Erastin and 2 μmol/L Fer-1 in the absence or presence of 100 ng/mL EGF for 24 h. Cell death were measured by flow cytometry.

(E-F) Parental Hep3B cells and the indicated clones with reconstituted expression of the WTAP S176A (left) or knock-in expression of GLS Mut (right) were treated with different doses of SAS (E) or Erastin (F) in the absence or presence of 100 ng/mL EGF for 24 h, and cell viability were measured by CCK-8 assay.

(G-I) Parental Hep3B cells and the indicated clones with knock-in expression of GLS Mut were stably transfected with WTAP shRNA and reconstituted with indicated WTAP proteins. The cells were treated with or without 20 μmol/L Erastin and 2 μmol/L Fer-1 for 24 h. Lipid ROS-positive cells (G) and cell death (H) were measured by flow cytometry, respectively. The cells were treated with different doses of Erastin (I) for 24 h, cell viability was measured by CCK-8 assay. Data are the mean ± SD, * *P* < 0.05; ***P* < 0.01; ****P* < 0.001 (two-tailed Student’s *t*-test).

Abbreviations: EGF, epidermal growth factor; KGA, kidney-type glutaminase; GAC, glutaminase C; GLS, glutaminase; WTAP, Wilms’ tumor 1-associated protein; SAS, Sulfasalazine; Fer-1, ferrostatin-1; WT, wild type; Mut, mutant; SD, standard deviation.


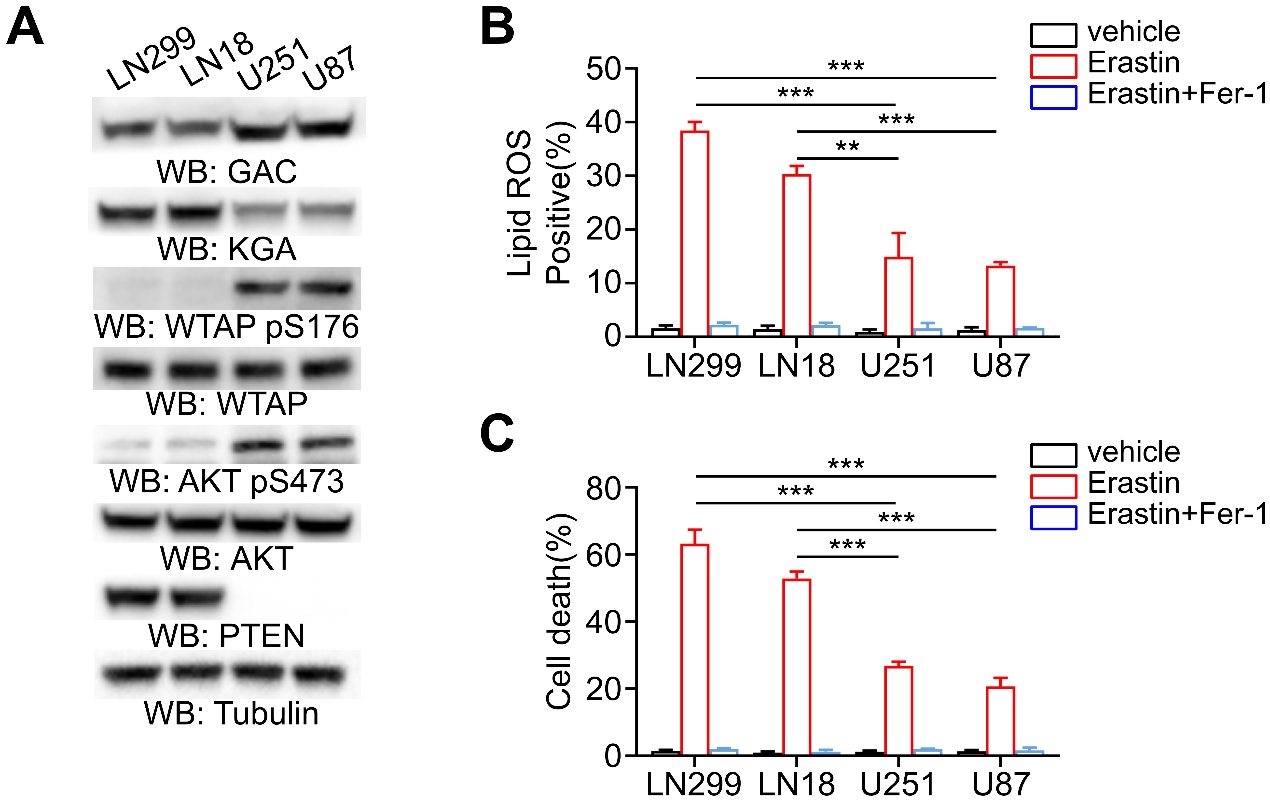


**Supplementary Figure S6. AKT-WTAP-GLS axis suppresses ferroptosis.**

(A) The whole cell lysates of LN229, LN18, U251, and U87 were harvested for western blotting analyses with the indicated antibodies.

(B-C) LN229, LN18, U251, and U87 cells were treated with or without 20 μmol/L Erastin combined with 2 μmol/L Fer-1 for 24 h. Lipid ROS-positive cells (B) and cell death (C) were measured by flow cytometry, respectively. Data are the mean ± SD (*n* = 3). ***P* < 0.01; ****P* < 0.001 (two-tailed Student’s *t*-test).

Abbreviations: WTAP, Wilms’ tumor 1-associated protein; KGA, kidney-type glutaminase; GAC, glutaminase C; Fer-1, ferrostatin-1; SD, standard deviation.


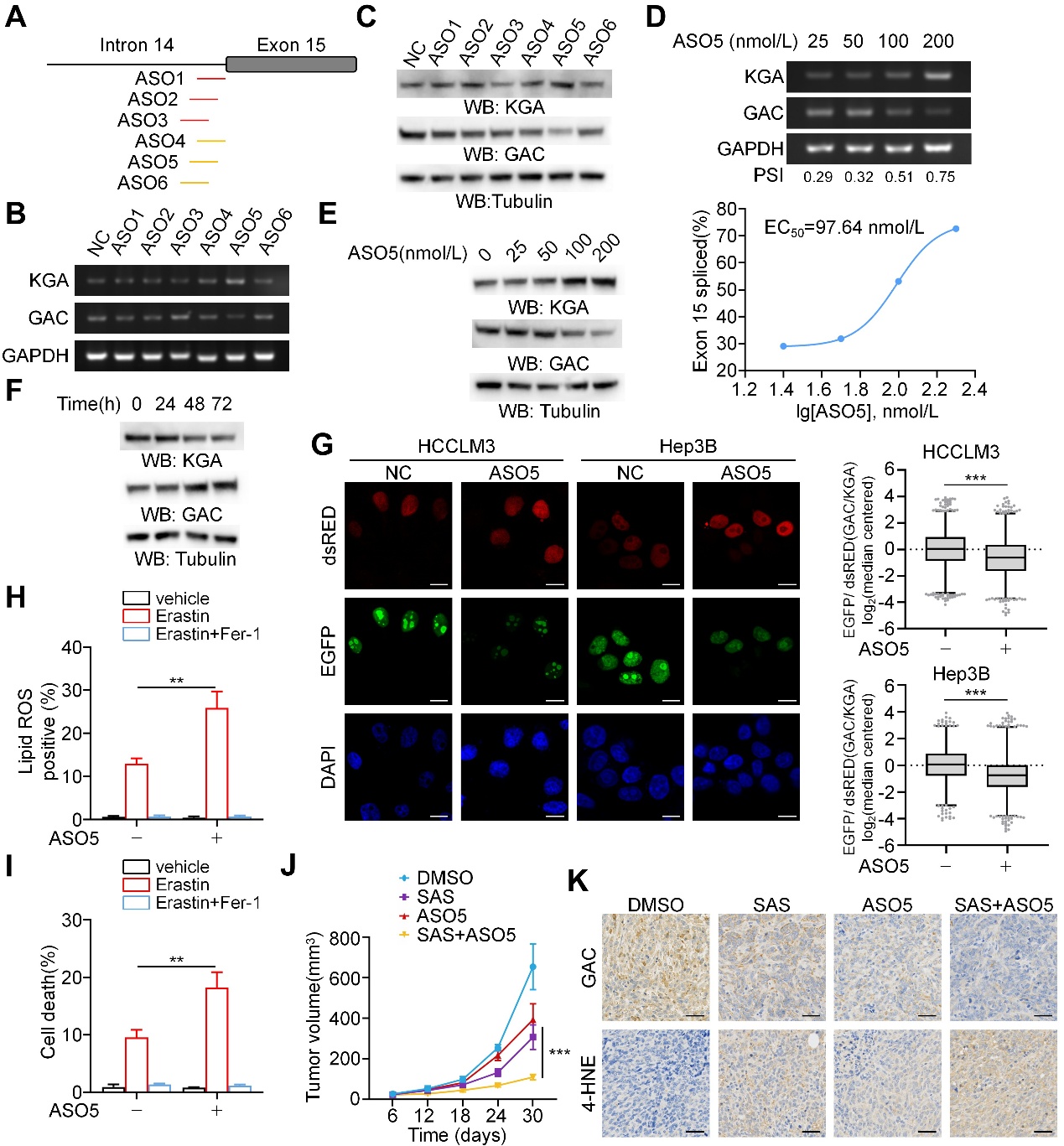


**Supplementary Figure S7. ASO-mediated GLS splicing alteration promotes ferroptosis of HCC.**

(A) Schematic diagram of ASO target sites on *GLS*.

(B) RT-PCR analysis of GLS splicing isoforms in response to ASOs.

(C) Western blotting of the GAC and KGA protein level in EGFRvIII-expressing HCCLM3 cells transfected with ASOs.

(D) Semi-quantitative RT-PCR analysis of the *GLS* AS pattern in EGFRvIII-expressing HCCLM3 cells transfected with ASO5. Dose-dependence curve of ASO5-treated EGFRvIII-expressing HCCLM3 cells showing increased skipping of exon 15 ([exon 15 skipped/exon 15 skipped + full length] × 100%) in relation to the log of the dose.

(E) Western blotting of the GAC and KGA protein level in EGFRvIII-expressing HCCLM3 cells transfected with different concentration of ASO5.

(F) KGA and GAC were measured by western blotting in EGFRvIII-expressing HCCLM3 cells treated with 200 nmol/L ASO5 for different times (0, 24, 48, and 72 h).

(G) Fluorescence microscopy images of EGFRvIII-expressing HCCLM3 and Hep3B cells transfected with the RG6 splicing reporter and the analysis of the EGFP/dsRED ratio. At least 1,835 independent cells evaluated for each group.

(H-I) EGFRvIII-overexpressing HCCLM3 cells transfected with ASO5 were treated with or without 20 μmol/L Erastin and 2 μmol/L Fer-1 for 24 h. Lipid ROS-positive cells (H) and cell death (I) were measured by flow cytometry.

(J) EGFRvIII-expressing HCCLM3 subcutaneous tumor growth treated with SAS or ASO5 (*n* = 6).

(K) IHC analyses of the indicated xenograft tumors from nude mice were performed with the indicated antibodies. Representative staining images are shown.

Data are the mean ± SD. ***P* < 0.01; ****P* < 0.001 (two-tailed Student’s *t*-test).

Abbreviations: KGA, kidney-type glutaminase; GAC, glutaminase C; GLS, glutaminase; EGFP, enhanced green fluorescent protein; dsRED, discosoma sp. red fluorescent protein; AS, alternative splicing; RT-PCR, reverse transcription PCR; SAS, Sulfasalazine; IHC, immunohistochemistry; 4-HNE, 4-hydroxynonenal; DMSO, dimethyl sulfoxide; SD, standard deviation.


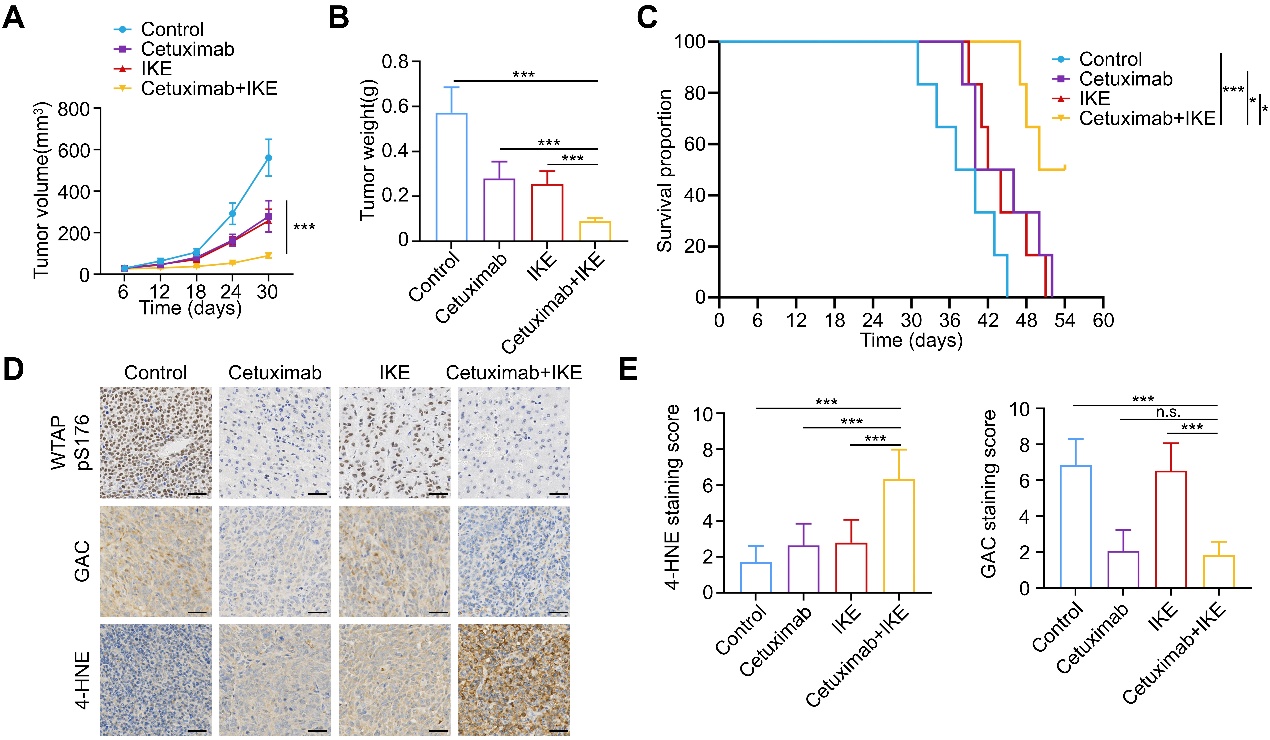


**Supplementary Figure S8. IKE combined with Cetuximab inhibits HCC tumor cell growth.**

(A-B) HCCLM3 cells were subcutaneously injected into 6-week-old male athymic nude mice (*n* = 6). When the tumor reached 50 mm^3^, the mice were assigned randomly into different treatment groups. IKE (30 mg/kg) was intraperitoneally injected every three days. Cetuximab was intraperitoneally injected (0.5 mg twice per week). Tumor volumes (A) and tumor weight (B) were calculated.

(C) Kaplan-Meier survival curves demonstrated significant difference between indicated cohorts (*n* = 6).

(D-E) IHC analyses of the control group, Cetuximab group, IKE group and Cetuximab + IKE group xenograft tumors from nude mice were performed with WTAP pS176, GAC and 4-HNE antibodies. (D) showed representative staining images from the xenograft tumors. The indicated staining scores in different groups were compared using two-tailed Mann-Whitney *U* test (E).

Data are the mean ± SD. **P*<0.05; ****P* < 0.001.

Abbreviations: IKE, imidazole ketone erastin; IHC, immunohistochemistry; WTAP, Wilms’ tumor 1-associated protein; GAC, glutaminase C; 4-HNE, 4-hydroxynonenal; SD, standard deviation.

**Supplementary Table S1. Detailed clinical features of 90 patients with HCC.**

| **Clinical feature** | | **Case No. (%)** |
| --- | --- | --- |
| Gender | Male | 80 (88.9) |
|  | Female | 10 (11.1) |
| Age (years) | ≤50 | 38 (42.2) |
|  | >50 | 52 (57.8) |
| AFP (ng/mL) | ≤20 | 25 (27.8) |
|  | >20 | 65 (72.2) |
| HBV | Yes | 78 (86.7) |
|  | No | 12 (13.3) |
| HCV | Yes | 4 (4.4) |
|  | No | 86 (95.6) |
| Tumor size (cm) | ≤5 | 68 (75.6) |
|  | >5 | 22 (24.4) |
| Vascular invasion | Yes | 21 (23.3) |
|  | No | 69 (76.7) |
| Grade | I-II | 59 (65.6) |
|  | III-IV | 31 (34.4) |

**Supplementary Table S2. RT-qPCR primers used in this study.**

| **Primer** | | **Sequence (5’-3’)** |
| --- | --- | --- |
| GAPDH | Forward | AGCCACATCGCTCAGACAC |
|  | Reverse | GCCCAATACGACCAATCC |
| KGA | Forward | ACTGAGCCCTGAAGCAGTTC |
|  | Reverse | CTGTCCTTGGGGAAAGGGTT |
| GAC | Forward | GCTGGTCTCCTCCTCTGGAT |
|  | Reverse | CCTCATTTGACTCAGGTGACA |
| GLS | Forward | CATGGGAGTCGACCTGAGTA |
|  | Reverse | GCTTTTCTCTCCCAGACTTTCC |

**Supplementary Table S3. The sequences of RNA probes used in this study.**

| **Probe** | **Sequence (5’-3’)** |
| --- | --- |
| M1-m^6^A | GUUGCUUGAA(m^6^A)CAACUAGCA |
| M1-A | GUUGCUUGAACAACUAGCA |
| M1-T | GUUGCUUGATCAACUAGCA |
| M2-m^6^A | UUCCUUUGGA(m^6^A)CCAUUGGAC |
| M2-A | UUCCUUUGGACCAUUGGAC |
| M2-T | UUCCUUUGGTCCAUUGGAC |

**Supplementary Table S4. The ssOND and primers used for sgRNA in this study.**

| **Primer** | **Sequence (5’-3’)** |
| --- | --- |
| GLS sgRNA | TTGAACAACTAGCATTCCTT |
| GLS PCR F | AAATTTTTGTTAGTTTTTAT |
| GLS PCR R | TTCTATTGCCACTAAAGACA |
| GLS Mut-ssOND | TACCATTTTATAGCTTGGACCTTTCTTCACTCAAGTGCACTAATAAATTATGTATGTTGCTTGATCAACTACCACAGCTTTGGACCATTGGACTATGAAAGTCTCCAACAAGAACTTGCTTTAAAAGAGA |
